# Phytochemical Profile and Anticancer Potential of *Helichrysum arenarium* Extracts on Glioblastoma, Bladder Cancer, and Breast Cancer Cells

**DOI:** 10.3390/ph18020144

**Published:** 2025-01-22

**Authors:** Ihsan Nalkiran, Hatice Sevim Nalkiran

**Affiliations:** Department of Medical Biology, Faculty of Medicine, Recep Tayyip Erdogan University, Rize 53020, Türkiye; ihsan.nalkiran@erdogan.edu.tr

**Keywords:** *Helichrysum arenarium*, pytochemicals, cytotoxicity, anticancer effects

## Abstract

Background/Objectives: Cancer is the second leading cause of death globally. Medicinal plants have emerged as fundamental sources of bioactive compounds with anticancer potential, largely attributed to their diverse secondary metabolites. This study aimed to investigate the cytotoxic effects of *Helichrysum arenarium* extracts from two distinct regions of Turkiye, Mersin, and Artvin, on cancerous (MDA-MB-231, RT4, T98G) and non-cancerous (ARPE-19, hGF) cell lines and to identify bioactive compounds responsible for these effects. Methods: *H. arenarium* plant extracts were prepared using ethanol and methanol as solvents, followed by lyophilization and dissolution in DMSO. The cytotoxic effects of the extracts were evaluated using Hoechst staining and MTS assays to assess cell viability. IC50 values and selectivity indices were calculated. Phytochemical composition was analyzed using Quadrupole Time-of-Flight mass spectrometry. Results: The ethanol extract from Mersin (HAE-M) demonstrated superior cytotoxicity, particularly against breast and bladder cancer cells, while showing minimal impact on non-cancerous cells. HAM-M, HAE-A, and HAM-A exhibited comparatively less potent effects. Phytochemical analysis of HAE-M identified 16 bioactive compounds, including Naringenin, Luteolin, and Quercitrin, known for their antioxidant and anticancer properties. Conclusions: These findings highlight the potential of *H. arenarium* extracts, particularly HAE-M, as a source of potent anticancer agents. This study is novel in its comprehensive analysis of different extraction methods and regional plant sources, combined with phytochemical profiling, to identify selective anticancer effects. Further investigations into the mechanisms of action of these extracts could contribute to the development of plant-derived anticancer therapies.

## 1. Introduction

Cancer remains one of the leading causes of morbidity and mortality worldwide. Among non-communicable diseases, cancer is the second leading cause of death after cardiovascular diseases. Among non-infectious diseases, cancer ranks as the second leading cause of death after cardiovascular diseases and is responsible for one in eight deaths globally [1,2,3,4]. Cancer remains one of the most significant diseases in modern medicine, primarily due to the ability of cancer cells to develop drug resistance and the incomplete understanding of the mechanisms underlying cancer progression. Despite significant advancements in cancer treatments and the application of molecular biology techniques over the past three decades, the global cancer burden continues to rise, and cancer remains one of the most lethal diseases worldwide. The primary strategies in cancer treatment include chemotherapy, surgery, and radiation therapy, often employed in combination or sequentially, along with targeted therapies, to reduce, resect, eliminate, or palliate tumors.

Chemotherapy is a routinely used method in cancer treatment. Cancer cells exhibit uncontrolled and sustained proliferation due to the loss of many regulatory functions that are typically present in normal cells. This characteristic renders cancer cells susceptible to chemotherapeutic drugs that target cellular division processes. Strategies aimed at controlling the initiation and progression of cancer are of critical importance in the development of effective therapeutic approaches. Nearly five decades of systemic drug discovery and development have culminated in the establishment of an extensive repertoire of efficacious chemotherapeutic agents.

Medicinal plants are of significant importance in cancer treatment due to their diverse chemical compounds, which serve as a valuable source for the discovery of novel active agents against cancer [5]. The development of anticancer drugs from plant-based sources requires the testing of cytotoxic compounds and the analysis of crude extracts from plants [6]. Therefore, the primary objective is to identify natural products with enhanced efficacy and greater specificity toward cancer cells [7].

Plants produce a diverse range of chemical compounds that are not directly associated with growth, referred to as secondary metabolites. Alkaloids, terpenoids, flavonoids, pigments, and tannins are key components of these compounds. Secondary metabolites exhibit various biological effects, including anti-inflammatory, anticancer, and contraceptive activities, as well as diverse impacts on hematopoietic cells [8], lipids [9], and cardiovascular systems [10].

The efficacy of various compounds as anticancer agents has been demonstrated, highlighting their role in the development of drugs used for cancer treatment [11,12]. Various therapies utilizing plant-derived products have been proposed for cancer treatment. Currently, plant-derived anticancer agents such as vinca alkaloids (vinblastine, vincristine, and vindesine), epipodophyllotoxins (etoposide and teniposide), taxanes (paclitaxel and docetaxel), and camptothecin derivatives (camptothecin and irinotecan) are utilized for chemotherapy purposes. The impact of plants on cancer treatment needs to be thoroughly investigated, and further studies should be conducted to yield positive outcomes.

*Helichrysum arenarium (H. arenarium)* is a medicinal aromatic plant that grows wild in Anatolia and is commonly used as an herbal tea [13,14]. *H. arenarium L. Moench* (commonly known as golden herb) is a medicinal aromatic plant with widespread distribution across Europe [13]. Pharmacological data also indicate that the flowers of *H. arenarium* are a rich source of various active compounds, including flavonoids, phenolics, polyphenols [13,15,16], flavones [17], essential oils [18], and coumarins [19]. These compounds exhibit a wide range of activities, including antioxidant [20], anticancer, immunostimulatory, anticoagulant [21,22], antibacterial, antiallergic, antitussive, bronchodilatory, and antiviral [23] effects. Although studies in the literature have reported that *H. arenarium* exhibits free radical scavenging [13], anti-atherosclerotic, anti-inflammatory [24], and lipid peroxidation-reducing [25] activities, no research has yet investigated the cytotoxic effects of its plant extracts on glioblastoma and breast cancer cells in relation to its anticancer properties. In this study, dried extracts of *H. arenarium* were prepared by first extracting the dried plant material using ethanol and methanol, followed by lyophilization, and subsequently dissolving the extracts in DMSO. The cytotoxic effects of the extract on cancer cells were investigated, and the extract with the most effective inhibitory activity on cancer cells was subjected to content analysis to identify its potential components.

## 2. Results

### 2.1. Cytotoxic Effects of Ethanol and Methanol Extracts of H. arenarium on Non-Cancerous and Cancer Cell Lines

**Figure 1:**
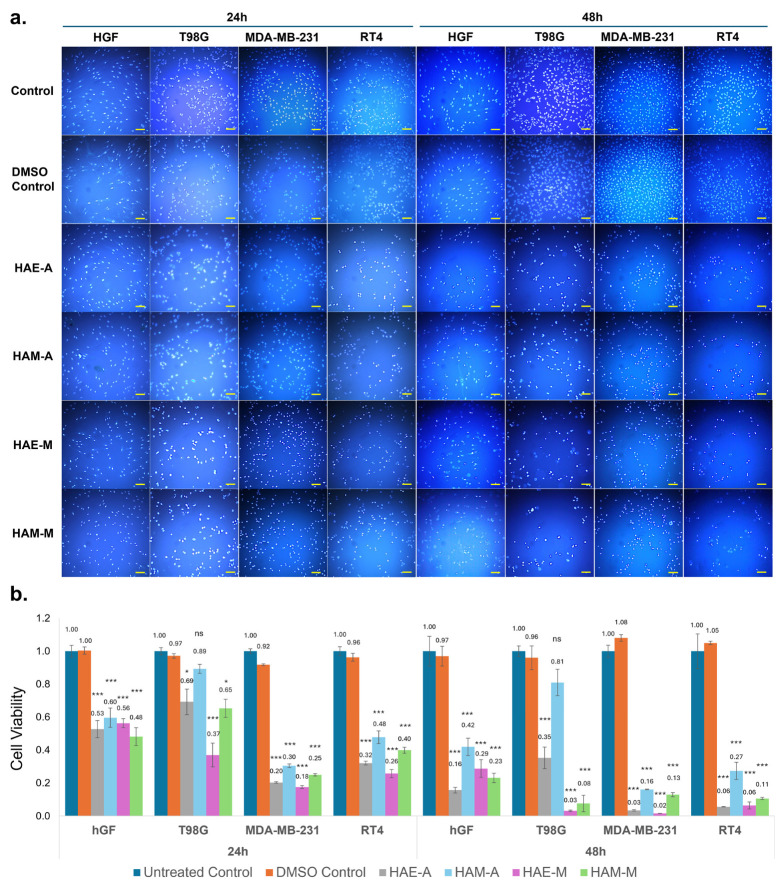
Hoechst staining and cell viability assessment using 2.5 µg/mL concentration of HAE-A, HAM-A, HAE-M, and HAM-M. (**a**) Hoechst staining of hGF, T98G, MDA-MB-231, and RT4 cells treated with 2.5 µg/mL of ethanol (HAE-A, HAE-M) and methanol (HAM-A, HAM-M) extracts for 24 and 48 h. (**b**) Cell viability measured by the MTS assay after 24 and 48 h. Statistical analyses (two-tailed *t*-test) were performed by comparing the results to the DMSO control group. Images were captured at 10× magnification. Scale bar represents 100 µm. Statistical significance was shown using asterisks; *: *p* < 0.05, and ***: *p* < 0.001, ns: non-significant.

The cytotoxic effects of 2.5 µg/mL concentration of ethanol extract of *H. arenarium* from Mersin (HAE-M) and methanol extract of *H. arenarium* from Mersin (HAM-M), ethanol extract of *H. arenarium* from Artvin (HAE-A), and methanol extract of *H. arenarium* from Artvin (HAM-A) were assessed on T98G, RT4, MDA-MB-231 cells, and non-cancerous hGF cells. The results are presented in Figure 1, with nuclear staining (normal nuclei and condensed nuclei) analyzed by Hoechst dye (Figure 1a), and the MTS assay used to quantitatively measure metabolic activity to assess overall cell viability and proliferation (Figure 1b).

Figure 1a demonstrates the effect of *H. arenarium* extracts on cell density by nuclear staining after 24 and 48 h of treatment. Treated cells exhibited a decreased number of cells based on the number of nuclei visualized using Hoechst staining, consistent with cytotoxicity in most cases. A noticeable but not severe decrease in the number of cells was observed with HAE-M and HAM-M treatments at both time points in T98G cells, indicated by visibly reduced nuclei, suggesting diminished proliferation and potential induction of cell death. This suggests that T98G cells are relatively more resistant to the extracts compared to other cancer cell lines but still susceptible to the stronger ethanol extract (HAE-M). RT4 cells exhibited a pronounced reduction in the number of cells based on nuclear staining in the group treated with HAE-M, particularly at 48 h, suggesting high sensitivity. HAE-M had the most significant impact, with a nearly complete reduction in the number of cells based on results obtained by Hoechst staining at 48 h in MDA-MB-231 cells. HAM-M also exhibited strong cytotoxicity, whereas HAE-A and HAM-A showed relatively weaker effects. The treatment with the extracts reduced the cell density in hGF cells, though the effects were less pronounced compared to cancer cell lines, suggesting some selectivity.

Figure 1b illustrates cell viability assessed by the MTS assay, confirming the dose- and time-dependent cytotoxic effects of *H. arenarium* extracts. The cytotoxic effects of HAE-M, HAM-M, HAE-A, and HAM-A extracts on non-cancerous, hGF, and cancerous (T98G, MDA-MB-231, and RT4) cell lines were evaluated using the MTS assay after 24 and 48 h of treatment. At 24 h, the non-cancerous hGF cells displayed moderate sensitivity to all extracts. Among the treatments, HAM-M caused the most significant reduction in cell viability (0.48, *p* < 0.001), followed by HAE-A (0.53, *p* < 0.001), HAE-M (0.56, *p* < 0.001), and HAM-A (0.60, *p* < 0.001) in hGF cells. In the T98G glioblastoma cells, HAE-M led to a substantial decrease in viability (0.37, *p* < 0.001), followed by HAM-M (0.65, *p* < 0.05), HAE-A (0.69, *p* < 0.05), and HAM-A (0.89, ns). The MDA-MB-231 triple-negative breast cancer cells were highly sensitive to all extracts, with HAE-M exhibiting the most potent effect (0.18, *p* < 0.001), followed by HAE-A (0.20, *p* < 0.001), HAM-M (0.25, *p* < 0.001), and HAM-A (0.30, *p* < 0.001). Similarly, in RT4 bladder cancer cells, HAE-M significantly reduced viability to 0.26 (*p* < 0.001), while HAE-A (0.32, *p* < 0.001), HAM-M (0.40, *p* < 0.001), and HAM-A (0.48, *p* < 0.001) showed moderate cytotoxic effects.

At 48 h, the cytotoxic effects of all extracts became more pronounced across both non-cancerous and cancerous cell lines, reflecting a clear time-dependent response. In hGF cells, viability further declined, with HAE-A reducing it to 0.16 (*p* < 0.001), followed by HAM-M (0.23, *p* < 0.001), HAE-M (0.29, *p* < 0.001), and HAM-A (0.42, *p* < 0.001). In T98G cells, HAE-M and HAM-M drastically reduced cell viability to 0.03 (*p* < 0.001) and 0.08 (*p* < 0.001), respectively, while HAE-A (0.35, *p* < 0.001), and HAM-A (0.81, ns) were less effective. MDA-MB-231 cells remained highly sensitive, with HAE-M causing a nearly complete loss of viability (0.02, *p* < 0.001), followed by HAE-A (0.03, *p* < 0.001), HAM-M (0.13, *p* < 0.001), and HAM-A (0.16, *p* < 0.001). In RT4 bladder cancer cells, HAE-M and HAE-A demonstrated strong effects, reducing viability to 0.06 (*p* < 0.001), with HAM-M (0.11, *p* < 0.001) and HAM-A (0.27, *p* < 0.001) showing progressively weaker cytotoxicity.

Overall, ethanol extracts (HAE-A and HAE-M) exhibited greater cytotoxicity among the extracts tested. HAE-M consistently showed the strongest cytotoxic activity, particularly against cancerous cell lines, with the most pronounced effects observed in MDA-MB-231 triple-negative breast cancer cells and RT4 bladder cancer cells. HAE-M also significantly reduced viability in T98G glioblastoma cells time-dependently. Notably, HAE-M demonstrated a stronger selectivity toward cancer cells, with comparatively less cytotoxic effects on non-cancerous cells, suggesting its potential as a safer therapeutic candidate. This selective cytotoxicity is crucial for cancer treatment strategies, aiming to minimize damage to healthy tissues while effectively targeting cancer cells.

The combined qualitative (Hoechst staining) and quantitative (MTS assay) analyses reveal that HAE-M is the most potent in reducing cancer cell viability, particularly in MDA-MB-231 and RT4 cells. This is supported by both the reduced number of visible nuclei observed in Hoechst staining and the significant reductions in metabolic activity measured by the MTS assay. HAM-M also displayed cytotoxic effects but was less effective than HAE-M across all cell lines. Notably, the cytotoxic effects of both extracts were more pronounced in cancer cells than in non-cancerous hGF cells, suggesting a level of selectivity. T98G glioblastoma cells exhibited the greatest resistance, with only moderate reductions in viability, highlighting the challenge of targeting glioblastoma with these extracts. In conclusion, these findings suggest that extracts of *H. arenarium*, particularly HAE-M, have promising selective cytotoxic activity against breast and bladder cancer cells, warranting further investigation into its therapeutic potential.

### 2.2. Dose- and Time-Dependent Cytotoxic Effects of HAE-M on Cancer and Non-Cancerous Cell Lines

**Figure 2:**
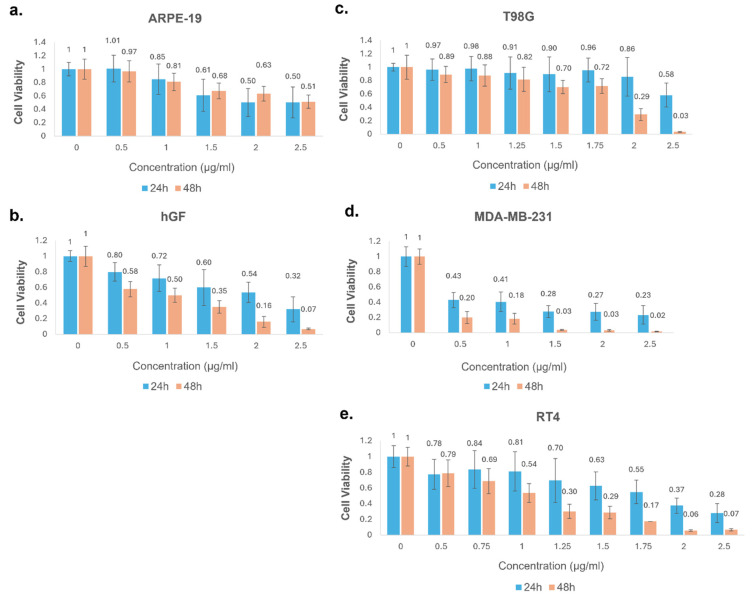
Dose- and time-dependent cytotoxic effects of HAE-M on cancerous and non-cancerous cell lines as assessed by the MTS assay. Cell viability was measured in (**a**) ARPE-19 (normal retinal epithelial cells), (**b**) hGF (human gingival fibroblasts), (**c**) T98G (glioblastoma), (**d**) MDA-MB-231 (triple-negative breast cancer), and (**e**) RT4 (bladder cancer) cell lines after 24 h and 48 h treatments with increasing concentrations of HAE-M extract (0–2.5 µg/mL). Data are presented as mean ± standard deviation (SD) from independent experiments. ARPE-19 and hGF cell lines were used as non-cancerous controls. Each dose normalized to the DMSO controls of each concentration. Concentration ‘0’ means untreated control.

HAE-M extract was further evaluated to determine dose-dependent effects on T98G, MDA-MB-231, and RT4 and non-cancerous ARPE-19 and hGF cells. To assess the dose- and time-dependent cytotoxicity of HAE-M, five cell lines were treated with concentrations ranging from 0 to 2.5 µg/mL for 24 and 48 h. The cell lines included ARPE-19, hGF, T98G, MDA-MB-231, and RT4. Cell viability was measured after each treatment period, as shown in Figure 2. For ARPE-19, cell viability gradually decreased with increasing extract concentrations and longer exposure times. At 24 h, cell viability remained above 0.8 (80%) relative to the control group for concentrations up to 1 µg/mL, with a more noticeable reduction at higher doses (0.61 at 1.5 µg/mL, 0.68 at 2 µg/mL, and 0.50 at 2.5 µg/mL). After 48 h, the cytotoxic effect was enhanced, with cell viability decreasing to 0.85 at 1 µg/mL, 0.63 at 2 µg/mL, and reaching 0.51 at 2.5 µg/mL. These results suggest that ARPE-19 cells exhibit a noticeable but not critical reduction in viability when treated with HAE-M, particularly with prolonged exposure. The hGF cells exhibited a more pronounced dose- and time-dependent decrease in viability than ARPE-19 cells. At 24 h, cell viability was reduced to 0.80 at 0.5 µg/mL, 0.72 at 1 µg/mL, and 0.54 at 2 µg/mL. After 48 h, this cytotoxic effect was further amplified, with viability dropping to 0.58 at 0.5 µg/mL, 0.50 at 1 µg/mL, and reaching 0.07 at 2.5 µg/mL. These findings indicate that hGF cells are sensitive to HAE-M, especially at higher concentrations and longer treatment durations.

T98G cells demonstrated moderate sensitivity to the HAE-M extract. At 24 h, cell viability remained high for concentrations up to 1.5 µg/mL, with only slight reductions (0.88 to 0.98). However, at higher concentrations, viability decreased to 0.70 at 1.75 µg/mL and 0.58 at 2.5 µg/mL. At 48 h, a more pronounced effect was observed, with viability dropping to 0.29 at 2 µg/mL and a significant reduction to 0.03 (3% viability) at 2.5 µg/mL. These results suggest that while T98G cells are initially more resistant, prolonged exposure to HAE-M significantly enhances cytotoxicity. The MDA-MB-231 cells were highly sensitive to HAE-M, showing a strong dose- and time-dependent reduction in viability. After 24 h, cell viability dropped sharply to 0.43 at 0.5 µg/mL and was nearly completely lost at 2 µg/mL (0.03, 3% viability) and 2.5 µg/mL (0.02, 2% viability). The 48-h treatment further intensified these effects, with viability reduced to 0.20 at 0.5 µg/mL and maintaining near-zero levels (0.02, 2% viability) at 2 µg/mL and above. These results indicate that MDA-MB-231 cells are highly susceptible to the cytotoxic effects of HAE-M, even at lower concentrations and shorter exposure times. RT4 cells also demonstrated significant dose- and time-dependent sensitivity to HAE-M. At 24 h, cell viability decreased gradually from 0.78 at 0.5 µg/mL to 0.55 at 1.75 µg/mL, with a marked reduction to 0.30 at 2.5 µg/mL. The 48-h exposure intensified these effects, with viability dropping from 0.37 at 1.5 µg/mL to as low as 0.07 (7% viability) at 2.5 µg/mL. This indicates that RT4 cells are moderately to highly sensitive to HAE-M, particularly with extended treatment times.

In summary, HAE-M exhibited a strong dose- and time-dependent cytotoxic effect across all tested cell lines, with the most substantial effects observed in cancer cell lines, particularly MDA-MB-231 breast cancer cells and RT4 bladder cancer cells. The extract also affected non-cancerous hGF and ARPE-19 cells, although to a lesser extent. T98G glioblastoma cells showed moderate sensitivity, with increased cytotoxicity following prolonged exposure. These findings suggest that HAE-M has potential selective cytotoxicity against cancer cells, particularly breast and bladder cancer, while also exhibiting a degree of cytotoxicity in non-cancerous cells at higher concentrations. Further studies are necessary to elucidate the specific bioactive components responsible for these effects and to explore their therapeutic potential for cancer treatment.

### 2.3. Dose- and Time-Dependent Cytotoxic Effects of HAM-M on Cancer and Non-Cancerous Cell Lines

**Figure 3:**
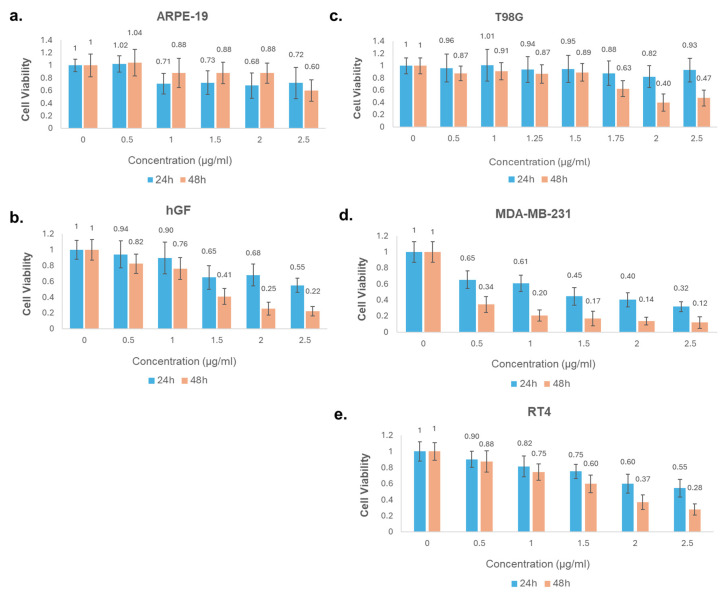
Dose- and time-dependent cytotoxic effects of HAM-M on cancerous and non-cancerous cell lines as assessed by the MTS assay. Cell Viability assessment of HAE-M extract at 24 and 48 h. Cell viability was measured in (**a**) ARPE-19 (normal retinal epithelial cells), (**b**) hGF (human gingival fibroblasts), (**c**) T98G (glioblastoma), (**d**) MDA-MB-231 (triple-negative breast cancer), and (**e**) RT4 (bladder cancer) cell lines after 24 h and 48 h treatments with increasing concentrations of HAM-M extract (0–2.5 µg/mL). ARPE-19 and hGF cell lines were used as non-cancerous controls. This table presents the results derived from four independent experiments. Data are presented as mean ± standard deviation (SD) from independent experiments. Each dose normalized to the DMSO controls of each concentration. Concentration ‘0’ means untreated control.

The cytotoxic effects of the HAM-M extract were evaluated on five cell lines, including ARPE-19, hGF, T98G, MDA-MB-231, and RT4. Cell viability was assessed at 24- and 48-h post-treatment with various concentrations of the extract (0.5–2.5 µg/mL), as shown in Figure 3. HAM-M demonstrated a slight dose-dependent reduction in the viability of ARPE-19 cells (Figure 3a). At the highest concentration (2.5 µg/mL), cell viability decreased to 72% after 24 h and to 60% after 48 h, indicating mild cytotoxicity in normal retinal epithelial cells over time. (Figure 3b). In hGF cells, the extract caused a marked dose- and time-dependent reduction in viability. At 2.5 µg/mL, viability dropped to 25% at 24 h and 22% at 48 h. T98G cells showed relative resistance to HAM-M extract (Figure 3c). Even at the highest tested concentration (2.5 µg/mL), viability remained above 90% at 24 h and 47% at 48 h, suggesting limited cytotoxic effects on this glioblastoma cell line. The MDA-MB-231 breast cancer cells were highly sensitive to the HAM-M extract (Figure 3d). At 1 µg/mL, cell viability decreased to 61% and 34% at 24 and 48 h, respectively. At 2.5 µg/mL, viability dropped to 12% after 48 h, highlighting strong anticancer activity against this cell line. In RT4 bladder cancer cells, the extract exhibited moderate cytotoxic effects in a time- and dose-dependent manner (Figure 3e). At 2.5 µg/mL, viability was reduced to 55% at 24 h and further to 28% at 48 h, indicating sensitivity to the HAM-M extract. In conclusion, the HAM-M extract exhibited differential cytotoxicity across the tested cell lines. It was most effective against the MDA-MB-231 cells, followed by RT4 cells, while showing limited effects on T98G cells. Non-cancerous cells (ARPE-19 and hGF) displayed moderate to significant sensitivity, suggesting potential off-target effects at higher concentrations.

### 2.4. Evaluation of Cytotoxic Effects of H. arenarium Extracts: IC50 Analysis in Cancer and Non-Cancerous Cell Lines

The cytotoxic effects of the HAE-M and HAM-M extracts were evaluated by determining the IC_50_ values in non-cancerous (ARPE-19, hGF) and cancerous (T98G, MDA-MB-231, RT4) cell lines at 24 and 48 h (Table 1). For HAE-M, the non-cancerous ARPE-19 cells exhibited high resistance, with an IC_50_ of 2.5 µg/mL at both 24 and 48 h, indicating limited cytotoxicity. The hGF cells demonstrated greater sensitivity over time, with the IC_50_ decreasing from 2.095 µg/mL at 24 h to 1 µg/mL at 48 h.

In cancerous cell lines, MDA-MB-231 triple-negative breast cancer cells were the most sensitive to HAE-M, with IC_50_ values of 0.215 µg/mL at 24 h and 0.125 µg/mL at 48 h, showing a strong time-dependent cytotoxic effect. RT4 bladder cancer cells also exhibited increased sensitivity over time, with IC_50_ values decreasing from 1.817 µg/mL at 24 h to 1.103 µg/mL at 48 h. For the T98G glioblastoma cell line, no IC_50_ was observed at 24 h, but an IC_50_ of 1.9 µg/mL was detected at 48 h, suggesting a delayed cytotoxic response.

For the HAM-M extract, no IC_50_ values (NA) were detected in ARPE-19, T98G, and RT4 cells at 24 h, indicating either low cytotoxicity or that the tested concentrations were insufficient to reduce viability by 50%. However, after 48 h, IC_50_ values were measurable in several cell lines: hGF cells showed an IC_50_ of 1.27 µg/mL, T98G cells had an IC_50_ of 2.14 µg/mL, MDA-MB-231 cells demonstrated increased sensitivity with an IC_50_ of 0.253 µg/mL, and RT4 cells had an IC_50_ of 1.777 µg/mL. Notably, ARPE-19 cells did not reach IC_50_ at either time point, indicating strong resistance to HAM-M.

Overall, the data reveal that both HAE-M and HAM-M extracts exert cytotoxic effects, with HAE-M displaying stronger and faster cytotoxicity, particularly against cancer cell lines. MDA-MB-231 cells were the most sensitive to both extracts, whereas ARPE-19 as a non-cancerous cell line consistently showed the highest resistance. In addition, the selectivity indices (SI) for HAE-M and HAM-M were calculated and are summarized in Table S2. The results highlight the differential cytotoxicity of the extracts across cancerous and non-cancerous cell lines. HAE-M demonstrated notable selectivity, particularly against the MDA-MB-231 breast cancer cell line. At 24 h, the SI was 11.63 relative to the ARPE-19 cells and 9.74 relative to the hGF cells, indicating strong selectivity. This selectivity increased at 48 h, reaching an SI of 20.0 for ARPE-19 and 8.0 for hGF, highlighting a time-dependent enhancement in cytotoxic selectivity toward breast cancer cells. In contrast, the selectivity of HAE-M against RT4 bladder cancer cells was moderate, with SI values of 1.38 (ARPE-19) and 1.15 (hGF) at 24 h, increasing to 2.27 (ARPE-19) at 48 h, which meets the threshold for selective cytotoxicity, although the SI decreased slightly to 0.91 relative to hGF cells. For T98G, HAE-M exhibited low selectivity at 48 h, with SI values of 1.32 (ARPE-19) and 0.53 (hGF), suggesting limited therapeutic potential against glioblastoma cells.

HAM-M displayed considerably weaker selectivity compared to HAE-M. For MDA-MB-231 cells, the SI was 1.89 (hGF) at 24 h, increasing to 5.02 at 48 h, indicating moderate selectivity, although no SI could be calculated against ARPE-19 due to the absence of cytotoxic effects (IC50 > 2.5 µg/mL). For RT4 cells, HAM-M showed poor selectivity, with no calculable SI at 24 h and an SI of 0.71 (hGF) at 48 h, suggesting non-selective cytotoxicity. Similarly, for T98G cells, the SI remained low at 0.59 (hGF), indicating poor selectivity.

Overall, the ethanol extract HAE-M demonstrated superior cytotoxic efficacy compared to the methanol extract HAM-M, as reflected by lower IC50 values across both cancerous and non-cancerous cell lines. In particular, the MDA-MB-231 breast cancer and RT4 bladder cancer cells were highly sensitive to HAE-M, displaying significantly lower IC50 values than those for HAM-M. HAE-M exhibits superior cytotoxic efficacy and selectivity against cancer cell lines, particularly the MDA-MB-231 cells, compared to the HAM-M. HAE-M showed strong, time-dependent cytotoxic effects with high selectivity indices, indicating its potential as a promising anticancer agent. Moderate selectivity was observed against RT4 cells, while limited selectivity was noted for T98G cells. Conversely, HAM-M displayed weaker cytotoxicity and lower selectivity across all tested cancer cell lines.

### 2.5. Phytochemical Composition of HAE-M Extract Reveals Bioactive Compounds with Antioxidant, Anti-Inflammatory, and Anticancer Potential

The qualitative analysis of the plant extract identified 16 metabolites based on their *m*/*z* values and relative abundance (Table 2). Gmelinol (C22H26O7) was the most abundant compound with an *m*/*z* of 401.16235 and an abundance of 194,746.47. This was followed by Athamantin (C24H30O7), with an *m*/*z* of 429.19356 and an abundance of 143,766.09. Naringenin (C15H12O5) was also identified, with an *m*/*z* of 271.06247 and an abundance of 31,658.24. PI(18:3(6Z,9Z,12Z)/18:3(9Z,12Z,15Z)) (C52H70O10) was detected with an *m*/*z* of 853.48977 and an abundance of 31,125.46, followed by 6-Gingesulfonic acid (C17H26O6S), which had an *m*/*z* of 417.15958 and an abundance of 29,045.23. Naringenin 7-O-glucoside (C21H22O10) showed an *m*/*z* of 433.11377 and an abundance of 23,330.15. Luteolin (C15H10O6) exhibited an *m*/*z* of 285.04163 and an abundance of 23,230.36. Isosalipurposide (C21H22O10) had an *m*/*z* of 433.11524 and an abundance of 22,815.72, while Pyrrhoxanthin (C39H48O6) displayed an *m*/*z* of 657.34406 and an abundance of 20,237.01. (14alpha,17beta,20S,22R)-14,20-Epoxy-17-hydroxy-1-oxowitha-3,5,24-trienolide (C28H36O5) was also identified with an *m*/*z* of 497.25637 and an abundance of 19,433.94. Neriantogenin (C23H32O4) had an *m*/*z* of 371.22419 and an abundance of 17,864.85, while Quercitrin (C21H20O11) showed an *m*/*z* of 447.09364 and an abundance of 17,096.13. 16-Deoxomexicanolide 16-methyl ether (C28H36O7) exhibited an *m*/*z* of 483.24107 with an abundance of 14,486.96, and Triterpenoid (C22H44N6O10) had an *m*/*z* of 551.30354 and an abundance of 9925.86. Finally, Aureusidin 6-rhamnoside (C21H20O10) displayed an *m*/*z* of 431.09919 with an abundance of 8179.1, and Esculentic acid (Phytolacca) (C30H46O6) had an *m*/*z* of 501.32453 and an abundance of 7271.06.

The qualitative analysis of the plant extract revealed a diverse profile of 16 bioactive metabolites with varying abundances, many of which are commonly associated with medicinal plants. Gmelinol emerged as the most abundant compound, followed by Athamantin and Naringenin, which are known for their bioactive properties. Other significant compounds, such as Luteolin, Naringenin 7-O-glucoside, Isosalipurposide, and Quercitrin, further highlight the richness of phenolic and flavonoid components within the extract.

## 3. Discussion

This study evaluated the cytotoxic effects of *H. arenarium* extracts, HAE-A, HAM-A, HAE-M, and HAM-M on cancer and non-cancerous cell lines, revealing significant differences in their potency and selectivity. The HAE-M exhibited the most pronounced cytotoxic effects, followed by the HAM-M, while HAE-A and HAM-A demonstrated potent however relatively weaker activity. The enhanced efficacy of HAE-M, particularly against MDA-MB-231 and RT4 cancer cells, highlights its potential as an anticancer candidate. This potency may be attributed to a higher content of bioactive compounds, such as flavonoids and phenolic derivatives, identified in the HAE-M extract. In comparison, HAM-M also exhibited significant cytotoxic effects, though it was less potent than HAE-M, suggesting that the ethanol extraction method may be more efficient in isolating cytotoxic secondary metabolites [26].

The use of both cancerous and non-cancerous cell lines in this study provided a comprehensive assessment of the selectivity and therapeutic potential of HAE-M and HAM-M. Hoechst staining was employed to observe the number of nuclei, specifically identifying features such as condensed nuclei and hallmarks of apoptosis, while the MTS assay quantitatively measured metabolic activity to reflect cell viability and proliferation. The combined analysis of these methods provided a comprehensive evaluation of the selective cytotoxicity of HAE-M and HAM-M, demonstrating their ability to preferentially target cancer cells while sparing normal cells. The cytotoxic effects were more pronounced in the cancer cell lines, particularly the MDA-MB-231 breast cancer and RT4 bladder cancer cells, compared to the non-cancerous hGF and ARPE-19 cells. This selectivity indicates the potential for *H. arenarium* extracts, particularly HAE-M, to target cancer cells with minimal impact on non-cancerous cells. This selectivity is a crucial characteristic of effective anticancer agents, as treatments must minimize damage to normal cells to reduce adverse effects in clinical applications. However, the moderate sensitivity of non-cancerous hGF cells to HAE-M and HAM-M at higher concentrations warrants further investigation to confirm their safety profile. The relatively lower toxicity in ARPE-19 and hGF cells supports the potential safety of the extracts. This approach indicates the promising selectivity of *H. arenarium* extracts as potential anticancer agents. In summary, HAE-M demonstrated superior cytotoxic activity compared to all other extracts, including HAM-M, HAE-A, and HAM-A.

Discovering the secondary metabolites of medicinal plants has led to various advancements in cancer treatment strategies [27]. The qualitative analysis of HAE-M revealed a diverse profile of plant-derived bioactive compounds that align with its traditional medicinal applications. Notably, Gmelinol, a lignan derivative, was identified as the most abundant compound. Lignans are well-established phenolic compounds with potent antioxidant and anti-inflammatory activities, contributing to plant defense mechanisms and showing promise for therapeutic use in inflammation-related diseases and cancer [28,29,30,31]. Athamantin, a coumarin derivative, ranked as the second most abundant compound. Coumarins are well-known plant secondary metabolites with diverse biological activities, including anticancer, anticoagulant, and antimicrobial effects [32]. The high abundance of Athamantin may suggest its role in the pharmacological potential of the extract.

Among the flavonoids, Naringenin, a subclass of flavanones, and its glycoside derivative Naringenin 7-O-glucoside, were prominently detected. Naringenin is extensively studied for its antioxidant, anti-inflammatory, and anticancer properties [33]. Naringenin is recognized as a novel phytomolecule due to its potent antioxidant, anti-inflammatory, and anticancer properties [33]. This phytomolecule has been shown to inhibit the migration and viability of TSGH-8301 bladder cancer cells by downregulating the AKT and MMP2 cell signaling pathways [34]. Its glycosylated form enhances solubility and bioavailability, underscoring the importance of flavonoid modifications in medicinal plants [35]. Similarly, Luteolin, a flavonoid from the flavone subclass, was identified and is known for its antitumor, anti-inflammatory, and neuroprotective effects [36,37,38]. The presence of multiple flavonoid glycosides, including Quercitrin, highlights the rich flavonoid composition of the extract, which plays a central role in its antioxidant and potential anticancer activity [39].

The analysis also detected phosphatidylinositol PI (18:3(6Z,9Z,12Z)/18:3(9Z,12Z,15Z)), a lipid molecule with two polyunsaturated fatty acid chains. These lipids are integral to cell membrane integrity and can influence signaling pathways associated with inflammation and apoptosis [40,41]. Additionally, the sulfur-containing compound 6-Gingesulfonic acid, derived from ginger, further supports the anti-inflammatory and secondary metabolite-enriched nature of the extract [42]. Secondary metabolites like Isosalipurposide, a flavonoid glycoside, and Pyrrhoxanthin, a carotenoid derivative, contribute to the antioxidant capacity of the extract and are associated with protective effects against oxidative stress and cancer progression [43,44,45].

Lower-abundance compounds such as 16-Deoxomexicanolide 16-methyl ether and Triterpenoid highlight the presence of oxygenated triterpenoids, which are known for their antimicrobial, anticancer, and hepatoprotective effects [46,47,48]. Triterpenoids have been widely recognized for their role in inducing apoptosis and inhibiting proliferation in cancer cells [49,50]. A limitation of this study is that the differentiation between Naringenin 7-O-glucoside and Isosalipurposide, sharing similar *m*/*z* values, was based solely on Q-TOF mass spectrometry through analysis of their ionization patterns, adduct formations, and retention times. While this method provides valuable insights, the absence of NMR spectroscopy limits definitive structural confirmation. Future studies will incorporate NMR analysis to enhance the accuracy and reliability of compound identification. Additionally, further studies will investigate the effects of these extracts on cancer cell behaviors, including migration, invasion, and apoptosis, to better understand their underlying mechanisms of action and therapeutic potential.

The overall compound profile of HAE-M reveals a dominance of flavonoids, lignans, and triterpenoids, all of which are well-documented plant secondary metabolites with therapeutic potential. The high abundance of flavonoids, particularly Naringenin, Luteolin, and Quercitrin, underscores the antioxidant and anticancer properties of the extract, aligning with its traditional use in inflammatory and oxidative stress-related disorders. Although purified compounds derived from medicinal plants have been extensively developed and are widely utilized in cancer therapies due to their targeted and potent anticancer effects with well-defined mechanisms of action, whole plant extracts offer unique therapeutic advantages. These advantages stem from their complex mixture of bioactive compounds, including flavonoids, phenolics, terpenoids, and alkaloids, which can work synergistically to influence multiple molecular pathways involved in cancer progression, metastasis, and drug resistance [51].

The synergistic and additive interactions between bioactive compounds within plant extracts often contribute to their enhanced therapeutic potential, which may not be replicated by single, isolated compounds [52]. In the case of *H. arenarium*, phytochemicals such as Naringenin and Quercitrin, identified through Q-TOF analysis, are well-documented for their anticancer, antioxidant, and anti-inflammatory properties [53,54]. Their combined presence in the extract likely contributes to the superior cytotoxicity observed, particularly with HAE-M, compared to methanol extracts. This synergistic interaction among bioactive compounds allows for the simultaneous modulation of multiple cancer-related pathways, offering a comprehensive therapeutic effect that is challenging to achieve with single-agent therapies. However, we recognize that plant extracts also present challenges in terms of standardization, dosing accuracy, and batch-to-batch consistency. Therefore, further investigations are needed to isolate and characterize individual bioactive components within *H. arenarium*, evaluate their synergistic interactions, and optimize their therapeutic efficacy through standardization.

The findings from this study suggest that HAE-M is a promising candidate for further pharmacological profiling and evaluations, particularly for anticancer and anti-inflammatory applications.

## 4. Methods

### 4.1. Extraction of H. arenarium Plant Extract

*H. arenarium*, sourced from a local herbal shop in Mersin (Southern Turkiye), was collected from the Toros area, while Artvin derived *H. arenarium* was obtained from Yusufeli, Artvin (North-East of Turkiye). Dried plant materials were used for the extraction to ensure consistency, stability, and reproducibility of the bioactive compounds. Although fresh specimens could also serve as a source of bioactive constituents, drying the plant material minimizes enzymatic degradation and microbial contamination. Additionally, it enhances the efficiency of the phytochemical extraction and allows for long-term storage. The aerial flower parts of *H. arenarium* were separated and ground into powder. The powdered material was extracted with a purity of 99.8% ethanol (1:20, *w*/*v*) and a purity of 99.9% methanol (1:20, *w*/*v*) by shaking at 125 rpm for 48 h at room temperature. The obtained extracts were filtered using medium-speed filters with a 110 mm diameter and the extract was mixed with dH_2_O (1:1, *v*/*v*), frozen at −80 degrees, and lyophilized for 48 h to completely remove the solvents. The remaining plant extracts in powdered form were weighed and dissolved in 100% DMSO to achieve a main stock concentration of 50 mg/mL. The samples were then diluted to different concentrations in a cell culture medium before application to the cells. The stock solutions were then serially diluted in the appropriate cell culture media to obtain a range (0.5–2.5 µg/mL) of working concentrations for the cytotoxicity assays. DMSO was used as the diluent, and the final DMSO concentration in all treatment and control wells was maintained below 0.01%, ensuring minimal solvent-related effects on the cells.

### 4.2. Culturing of Cells

This study utilized RT-4 (human recurrent, differentiated transitional carcinoma of the bladder), generously provided by Prof. Devrim Gozuacik (Koc University, Istanbul, Turkiye); T98G (human glioblastoma cells), purchased from the American Type Culture Collection (ATCC); MDA-MB231 (human triple-negative metastatic breast cancer cells), kindly provided by Prof. Selcen Celik Uzuner, Blacksea Technical University, Turkey; ARPE-19 (human normal retinal pigment epithelial cells), kindly provided by Prof. Saliha Eksi (Recep Tayyip Erdogan University, Rize, Turkiye); and hGF (PCS-201-018, human gingival fibroblast cells), purchased from ATCC. All cells were cultured in cell media containing 10% Fetal Bovine Serum (FBS) (Sigma-Aldrich, St Louis, MO, USA) and 1% Penicillin/Streptomycin. The RT-4, MDA-MB231, and ARPE-19 cells were grown in RPMI 1640 medium, the hGF cells were cultured in MEM, and the T98G cells were cultured in high-glucose DMEM. All cultures were maintained in a cell culture incubator at 37 °C with 5% CO_2_. Prior to use, all solutions (culture medium and stock solutions of the *H. arenarium* extracts) used in the culture process were sterilized through 0.22 µm filters. According to the experimental objectives, the cultures were conducted in 6- or 96-well plates or in sterile flasks of 25–75 cm^2^. To comprehensively evaluate the selectivity of the *H. arenarium* extracts, both cancerous and non-cancerous cell lines were utilized in this study. This dual-cell model system was employed to assess whether the extracts selectively induce cytotoxicity in cancer cells without significantly affecting non-cancerous cells. This comparative analysis provides critical insights into the therapeutic potential and safety profile of the extracts, aligning with standard practices in anticancer drug screening to identify compounds with high tumor specificity and minimal off-target toxicity.

### 4.3. Treatment with H. arenarium Extract, Hoechst Staining, and Cytotoxicity Assays

Cells were seeded in flat bottom 96-well plates at a density of 1 × 10^4^ cells per well in 100 µL of medium for each cell line. After an overnight incubation to facilitate cell attachment, the plant extracts were administered. The cytotoxic effects of DMSO-dissolved forms of the previously lyophilized HAE-M, HAM-M, HAE-A, and HAM-A were assessed. The DMSO-dissolved lyophilized plant samples were applied initially at 2.5 µg/mL dose and further in a series of serially diluted concentrations within a specified range for 24 h and 48 h. For the ARPE-19, hGF, MDA-MB-231, and RT4 cell lines, the concentrations used were 0.5, 1, 1.5, 2, and 2.5 µg/mL. For the T98G cell line, a slightly broader range of concentrations was applied, including 0.5, 1, 1.25, 1.5, 1.75, 2, and 2.5 µg/mL. The same concentration of 2.5 µg/mL was consistently used for both Hoechst staining and the initial MTS assays across all experimental groups. This concentration was selected based on standard practices in cytotoxicity screening, where an upper limit of 2.5 µg/mL is often used to identify potential cytotoxic effects while minimizing the risk of non-specific toxicity. To control for any potential solvent effects, DMSO control groups were included for each concentration, where cells were treated with the same amount of DMSO present in the corresponding extract dilutions. For each experimental condition, parallel cultures were prepared and treated identically for both Hoechst staining and MTS assays. Importantly, these assays were conducted on separate plates to prevent any potential interference between fluorescence imaging and metabolic activity measurements. Following incubations (24 h and 48 h), the live cells were stained with Hoechst dye to visualize nuclei. A Hoechst working solution was prepared at 1–10 µg/mL in a pre-warmed culture medium. The dye solution was added to the cells and incubated at 37 °C for 10 min in a humidified CO₂ incubator. Following incubation, cells were gently washed once with pre-warmed PBS. Fluorescence images were captured immediately using an inverted fluorescence microscope (DM-IL-LED, Leica Microsystems, Wetzlar, Germany).

Cell proliferation was assessed using the (3-(4,5-dimethylthiazol-2-yl)-5-(3-carboxymethoxyphenyl)-2-(4-sulfophenyl)-2H-tetrazolium) (MTS) assay with MTS (4.2 mg/mL) (Promega, Madison, WI, USA) and phenazine methosulfate (PMS) (0.92 mg/mL) (Sigma Aldrich, St Louis, MO, USA) dissolved in DPBS without Mg^2+^ and Ca^2+^. For a 96-well plate, a mixture was prepared by combining 8.8 mL of medium, 2.2 mL of MTS solution, and 110 µL of PMS solution, and the medium in each well was aspirated, and 100 µL of the medium/MTS/PMS mixture was added. After a 2-h incubation at 37 °C, absorbance was measured at 492 nm using a Multiskan GO microplate reader (Thermo Fisher, Waltham, USA), and IC50 values were calculated using the IC50 toolkit (ic50.tk). The MTS assay was conducted after 24 and 48 h of incubation with the extracts. Untreated cells served as negative controls and DMSO-treated cells were included as solvent controls to account for any solvent-related effects. Additionally, a blank well containing only the MTS solution without cells was included to correct for background absorbance. The absorbance of the blank solution was subtracted from all absorbances obtained from the control and treatment groups. The relative cell viability was calculated by dividing the absorbance value of the treated cells by the absorbance value of the control cells treated with DMSO. In other words, the absorbance measured from the treated cells reflects their metabolic activity, which correlates with the number of viable cells. This value was then compared to the absorbance of the untreated or DMSO-treated control cells. A result of 1 signifies that the treatment had no effect on viability, while values less than 1 indicate a reduction in cell viability.

The selectivity index (SI) was calculated to evaluate the ability of HAE-M and HAM-M to selectively target cancer cells while minimizing cytotoxic effects on non-cancerous cells. The SI was determined by dividing the IC_50_ values of non-cancerous cells (ARPE-19 and hGF) by those of cancerous cells (MDA-MB-231, RT4, and T98G), with higher SI values indicating greater selectivity for cancer cells. An SI value greater than 2 is generally considered indicative of selective cytotoxicity toward cancer cells.

### 4.4. Q-TOF Analysis for Compounds

Plant samples were extracted using a solvent appropriate for the target phytochemicals, such as methanol or ethanol to enhance solubility and compound recovery. The extract was then concentrated under reduced pressure and filtered to eliminate particulates, ensuring a clear solution (e.g., methanol or acetonitrile with 0.1% formic acid) suitable for injection into the liquid chromatography (LC) system (See Table S1).

The analysis was conducted on an Agilent 6200 series Time-of-Flight (TOF) mass spectrometer (Agilent Technologies, Santa Clara, CA, USA) coupled with a 6500 series Quadrupole Time-of-Flight (Q-TOF) mass spectrometer, using an electrospray ionization (ESI) source in negative ion mode (M−H)−(M-H)^−(M−H)−. Instrument parameters were optimized for phenolic and acidic compound detection, including the fragmentor voltage, which was adjusted to maximize ionization efficiency and the collision energy tailored to promote ideal fragmentation. This configuration allowed for precise mass analysis and structural elucidation through ion fragmentation patterns. Data processing was achieved using the qualitative analysis software version B.08.00, which includes comprehensive signal-to-noise checking and compound identification through database matching. The software generated a peak list for all detected compounds, with matches scored by a confidence level index (Score (DB)) indicating the similarity to reference compounds in the database. For each detected compound, the molecular formula (based on accurate mass and isotopic distribution), *m*/*z* values, quality scores, database hit counts, detected ion types (e.g., (M-H)−, (M+CH3COO)−), and abundance were systematically recorded. Identified compounds included various bioactive phytochemicals, such as Naringenin, Luteolin, and Quercitrin, each presented with its specific mass spectral features. To confirm the identity and structure of each compound, MS/MS fragmentation analysis was conducted, providing structural details and supporting the preliminary identifications. Relative quantification was achieved by examining the abundance values, allowing for the comparison of phytochemical concentrations within the extract. Calibration with internal reference mass (IRM) standards and control samples was performed to ensure mass accuracy, with IRM calibration status checked periodically to confirm instrument consistency throughout the data acquisition phase. The resulting dataset comprised detailed information on each identified compound, with abundance and *m*/*z* values presented in comprehensive tables. The chromatographic profile, retention time, peak area, and spectral data of each compound were documented to provide a robust overview of the phytochemical composition of the plant extract.

### 4.5. Statistical Analysis

All experiments were conducted in triplicate and repeated independently at least three times to ensure reproducibility. Data are presented as the mean ± standard deviation (SD). Statistical analysis was performed using a two-tailed unpaired Student’s *t*-test to compare the treatment groups with the DMSO control group. A *p*-value of less than 0.05 was considered statistically significant. The levels of significance were indicated as follows: *p* < 0.05 (*), *p* < 0.01 (**), and *p* < 0.001 (***). Statistical analyses were performed using ‘*t* test calculator tool’ available at https://www.graphpad.com/quickcalcs/ttest1 (accessed on 10 December 2024).

## 5. Conclusions

This study highlights the cytotoxic potential of *H. arenarium* extracts, particularly HAE-M, which demonstrated superior activity against cancer cell lines, including MDA-MB-231 breast cancer and RT4 bladder cancer cells. The selective cytotoxicity of HAE-M, combined with its reduced effects on non-cancerous cells, underscores its promise as a candidate for anticancer therapy. The qualitative analysis revealed a diverse phytochemical profile, including high concentrations of bioactive compounds such as Gmelinol, Athamantin, Naringenin, and Luteolin, which are associated with antioxidant, anti-inflammatory, and anticancer activities. These findings support the therapeutic potential of *H. arenarium*, emphasizing the importance of plant-derived bioactive compounds in developing novel cancer treatments. Future research should prioritize the detailed investigation of the molecular mechanisms underlying the observed anticancer effects of the HAE-M extract.

## Figures and Tables

**Table 1:** IC50 values (µg/mL) of HAE-M and HAM-M extracts in cancerous and non-cancerous cell lines at 24 and 48 h.

		ARPE-19 (µg/mL)	hGF (µg/mL)	T98G (µg/mL)	MDA-MB-231 (µg/mL)	RT4 (µg/mL)
HAE-M	24 h	2.5	2.095	NA	0.215	1.817
	48 h	2.5	1	1.9	0.125	1.103
HAM-M	24 h	NA	2.5	NA	1.325	NA
	48 h	NA	1.27	2.14	0.253	1.777

This table presents the results derived from four independent experiments. NA: not applicable or IC_50_ not reached within the tested concentration range.

**Table 2:** Compound analysis of HAE-M.

Number	Name	*m*/*z*	Abund/(%)	Formula	Ion	Score (DB)	Hits (DB)
1	Gmelinol	401.16235	194,746.47 (29.90)	C22 H26 O7	(M-H)−	78.15	10
2	Athamantin	429.19356	143,766.09 (22.07)	C24 H30 O7	(M-H)−	87.91	10
3	Naringenin	271.06247	31,658.24 (4.86)	C15 H12 O5	(M-H)−	88.47	10
4	PI(18:3(6Z,9Z,12Z)/18:3(9Z,12Z,15Z))	853.48977	31,125.46 (4.77)	C52 H70 O10	(M-H)−	90.23	10
5	6-Gingesulfonic acid	417.15958	29,045.23 (4.46)	C17 H26 O6 S	(M+CH3COO)−	90.29	1
6	Naringenin 7-O-glucoside	433.11377	23,330.15 (3.58)	C21 H22 O10	(M-H)−	94.88	10
7	Luteolin	285.04163	23,230.36 (3.56)	C15 H10 O6	(M-H)−	89.55	10
8	Isosalipurposide	433.11524	22,815.72 (3.50)	C21 H22 O10	(M-H)−	91.13	10
9	6-Gingesulfonic acid	403.14215	20,828.5 (3.19)	C17 H26 O6 S	(M+HCOO)−	91.89	1
10	Pyrrhoxanthin	657.34406	20,237.01 (3.10)	C39 H48 O6	(M+HCOO)−	92.58	1
11	(14alpha,17beta,20S,22R)-14,20-Epoxy-17-hydroxy-1-oxowitha-3,5,24-trienolide	497.25637	19,433.94 (2.98)	C28 H36 O5	(M+HCOO)−	85.51	3
12	Neriantogenin	371.22419	17,864.85 (2.74)	C23 H32 O4	(M-H)−	74.81	10
13	Quercitrin	447.09364	17,096.13 (2.62)	C21 H20 O11	(M-H)−	91.52	10
14	6-Gingesulfonic acid	417.15803	16,142.94 (2.47)	C17 H26 O6 S	(M+CH3COO)−	91.76	1
15	16-Deoxomexicanolide 16-methyl ether	483.24107	14,486.96 (2.22)	C28 H36 O7	(M-H)−	86.47	5
16	Triterpenoid	551.30354	9925.86 (1.52)	C22 H44 N6 O10	(M-H)−	92.95	3
17	Aureusidin 6-rhamnoside	431.09919	8179.1 (1.25)	C21 H20 O10	(M-H)−	78.93	10
18	Esculentic acid (Phytolacca)	501.32453	7271.06 (1.11)	C30 H46 O6	(M-H)−	85.51	5

Synthetic and non-plant-derived compounds were excluded from the preliminary analysis. The reported percentages were calculated based on the relative abundance of the identified bioactive, plant-derived compounds in the HAE-M extract. The presence of 6-Gingesulfonic acid with different *m*/*z* values in the table likely indicates the detection of different ionized forms or adducts of the same compound during the mass spectrometry analysis. (M-H)−: Deprotonated ion, (M+CH3COO)−: Acetate adduct ion, (M+HCOO)−: Formate adduct ion. See Supplementary File.

## Data Availability

The data presented in this study are available on request from the corresponding author.

## Supplementary Materials

The following supporting information can be downloaded at: https://www.mdpi.com/article/10.3390/ph18020144/s1, Table S1: Instrument and analytical parameters.; Table S2: Selectivity index of HAE-M and HAM-M on cancer cell lines; qualitative analysis report.
