# Supplementary material for: Phytochemical Profile and Anticancer Potential of Helichrysum arenarium Extracts on Glioblastoma, Bladder Cancer, and Breast Cancer Cells
Source: Pharmaceuticals (Basel). 2025 Jan 22;18(2):144. doi: 10.3390/ph18020144 (PMC11859872; doi:10.3390/ph18020144)
Supplement: Supplementary file 1 [file pharmaceuticals-18-00144-s001.zip › pharmaceuticals-3412281-supplementary.pdf]

Table S1. Instrument and Analytical Parameters.

Parameter	Details
Instrument	Agilent 6530B Q-TOF Mass Spectrometer
Ionization Mode	Dual Electrospray Ionization (ESI)
Acquisition Mode	AutoMS^2
Column	Poroshell 120 EC-C18 (3 × 50 mm, 2.7 µm)
Column Temperature	30°C
Mobile Phase (Solvent A)	90% H_2O with 0.1% Formic Acid
Mobile Phase (Solvent B)	10% Acetonitrile/Methanol
Flow Rate	0.4 mL/min
Injection Volume	1.0 µL
Gradient Program	0.5 min: A 90% / B 10% → 4 min: A 50% / B 50% → 9 min: A 5% / B 95%
Nebulizer Pressure	35 psi
Gas Flow Rate	10 L/min
Gas Temperature	325°C
VCap Voltage	3000 V
Fragmentor Voltage	200 V
Skimmer Voltage	65 V
Octopole RF	750 V
MS Scan Range	m/z 50–1200
MS/MS Scan Rate	2 spectra/sec
Collision Energy (CE)	10, 20, 40 eV
Isolation Width	Medium (~4 amu)
Retention Time (RT)	Variable based on compound elution

Table S2. Selectivity Index of HAE-M and HAM-M on cancer cell lines.

	**Cancer Cell Line**	**24h SI (ARPE-19)**	**24h SI (hGF)**	**48h SI (ARPE-19)**	**48h SI (hGF)**
**HAE-M**	MDA-MB-231	11.63	9.74	20.0	8.0
	RT4	1.38	1.15	2.27	0.91
	T98G	NA	NA	1.32	0.53
**HAM-M**	MDA-MB-231	NA	1.89	NA	5.02
	RT4	NA	NA	NA	0.71
	T98G	NA	NA	NA	0.59

Qualitative Analysis Report

**Data Filename**	AU1_Neg.d	**Sample Name**	AU1
**Sample Type**	Sample	**Position**	P1-A3
**Instrument Name**	Instrument 1	**User Name**	
**Acq Method**	Phenolic_Compounds-ESI_Pos.m	**Acquired Time**	8/10/2023 12:08:43 PM
**IRM Calibration Status**	Success	**DA Method**	SignalToNoiseCheckout.m
**Comment**			

**Sample Group**		**Info.**	
**Stream Name**	LC 1	**Acquisition SW Version**	6200 series TOF/6500 series Q-TOF B.08.00 (B8058.0)

User Chromatograms

Fragmentor Voltage 90 **Collision Energy** 0 **Ionization Mode** ESI

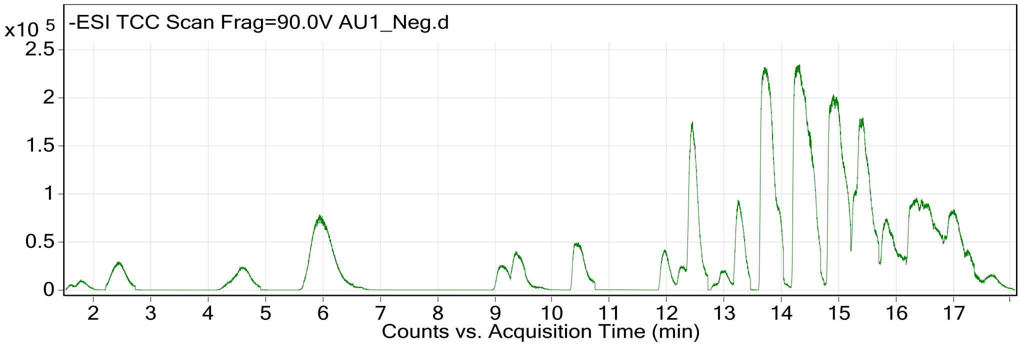

Compounds

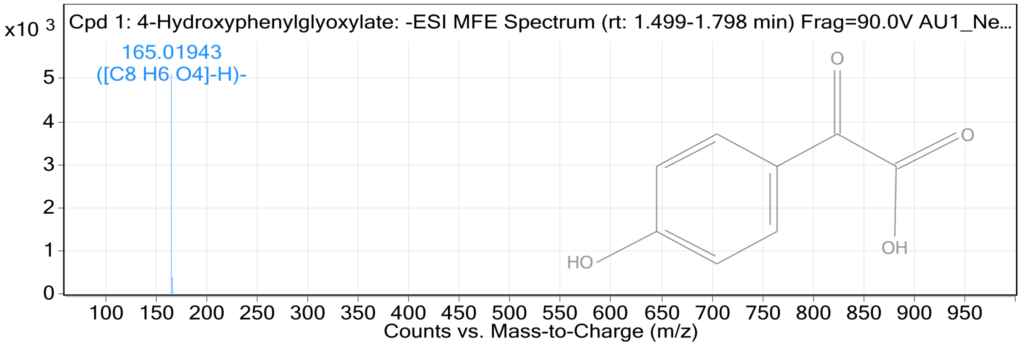

Peak List

m/z	z	Abund	Name	Formula	Ion	Score (DB)	Hits (DB)
165.01943	-1	5102.55	4-Hydroxyphenylglyoxylate	C8 H6 O4	(M-H)-	86.49	7
166.0222	-1	384.98		C8 H6 O4	(M-H)-		

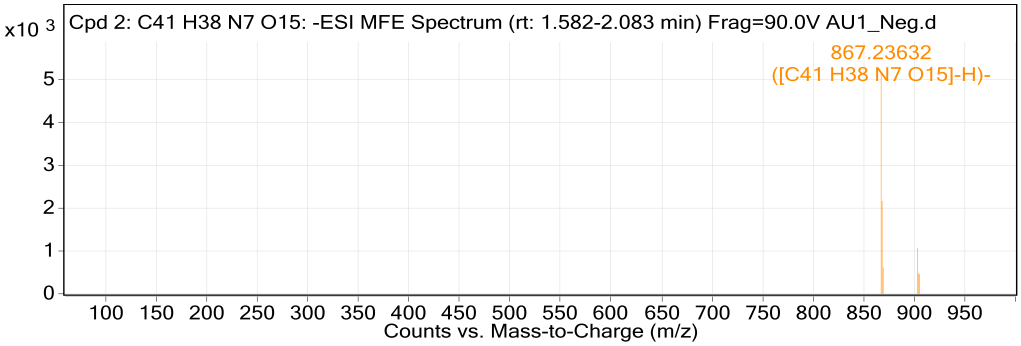

Peak List

m/z	z	Abund	Formula	Ion

867.23632	-1	5125.33	C41 H38 N7 O15	(M-H)-
868.23913	-1	2170.1	C41 H38 N7 O15	(M-H)-
869.23754	-1	612.64	C41 H38 N7 O15	(M-H)-
903.20863	-1	1074	C41 H38 N7 O15	(M+Cl)-
904.20952	-1	475.87	C41 H38 N7 O15	(M+Cl)-
905.206	-1	460.88	C41 H38 N7 O15	(M+Cl)-

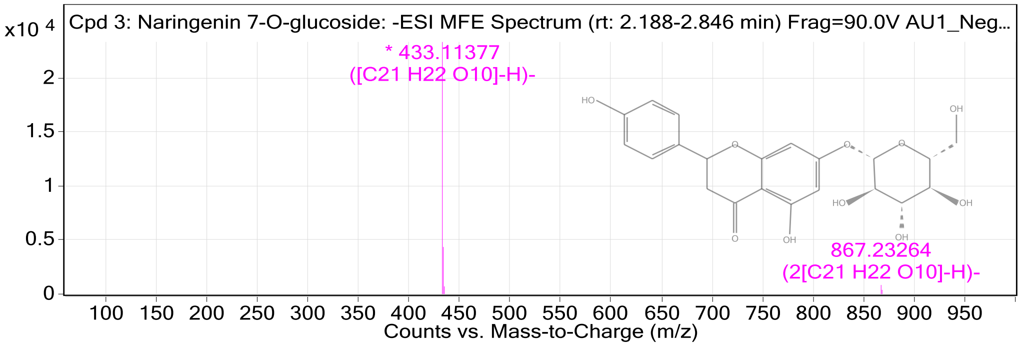

Peak List

m/z	z	Abund	Name	Formula	Ion	Score (DB)	Hits (DB)
433.11377	-1	23330.15	Naringenin 7-O-glucoside	C21 H22 O10	(M-H)-	94.88	10
434.11805	-1	4355.5		C21 H22 O10	(M-H)-		
435.11946	-1	708.59		C21 H22 O10	(M-H)-		
867.23264	-1	839.28		C21 H22 O10	(2M-H)-		
868.232	-1	392.16		C21 H22 O10	(2M-H)-		

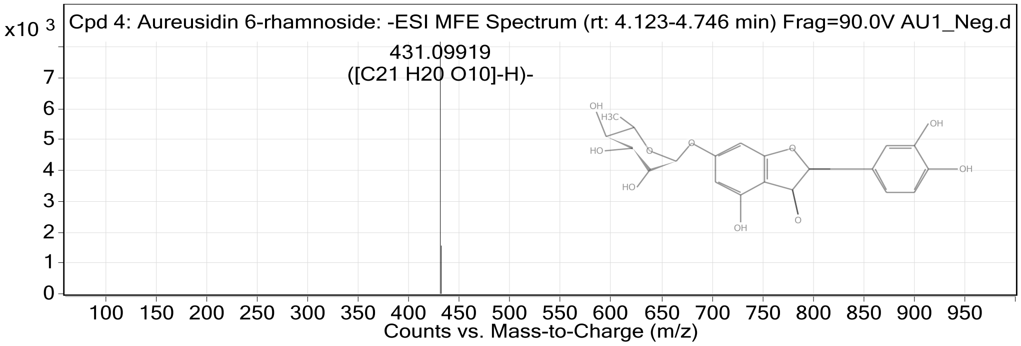

Peak List

m/z	z	Abund	Name	Formula	Ion	Score (DB)	Hits (DB)
431.09919	-1	8179.1	Aureusidin 6-rhamnoside	C21 H20 O10	(M-H)-	78.93	10
432.10232	-1	1564.14		C21 H20 O10	(M-H)-		

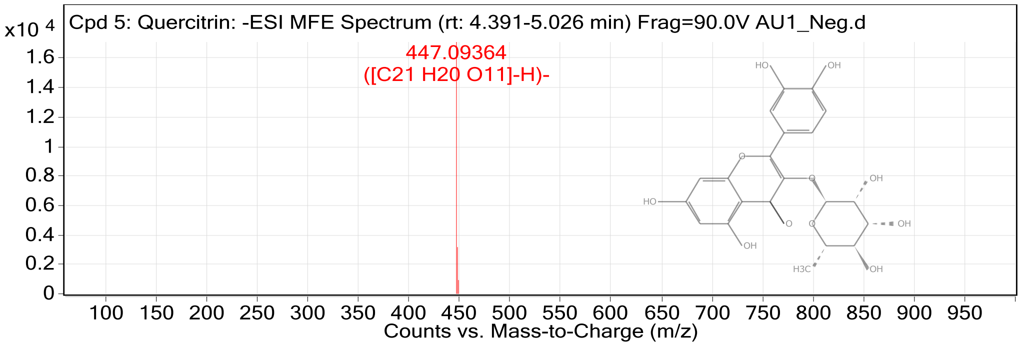

Peak List

m/z	z	Abund	Name	Formula	Ion	Score (DB)	Hits (DB)
447.09364	-1	17096.13	Quercitrin	C21 H20 O11	(M-H)-	91.52	10
448.09773	-1	3172.23		C21 H20 O11	(M-H)-		
449.10363	-1	935.83		C21 H20 O11	(M-H)-		

Qualitative Analysis Report

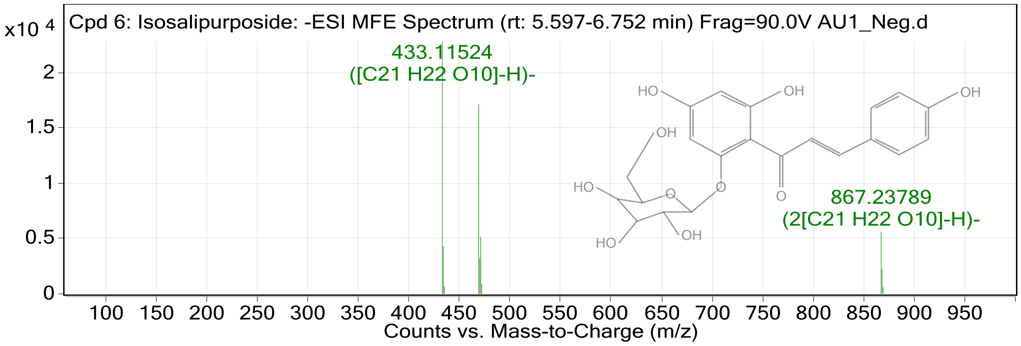

Peak List

m/z	z	Abund	Name	Formula	Ion	Score (DB)	Hits (DB)
433.11524	-1	22815.72	Isosalipurposide	C21 H22 O10	(M-H)-	91.13	10
434.11948	-1	4294.7		C21 H22 O10	(M-H)-		
435.12005	-1	705.94		C21 H22 O10	(M-H)-		
469.09224	-1	17143.47		C21 H22 O10	(M+Cl)-		
470.09626	-1	3243.74		C21 H22 O10	(M+Cl)-		
471.09118	-1	5162.6		C21 H22 O10	(M+Cl)-		
472.09291	-1	902.51		C21 H22 O10	(M+Cl)-		
473.08633	-1	2.68		C21 H22 O10	(M+Cl)-		
867.23789	-1	5589.96		C21 H22 O10	(2M-H)-		
868.24044	-1	2284.89		C21 H22 O10	(2M-H)-		
869.23987	-1	604.67		C21 H22 O10	(2M-H)-		

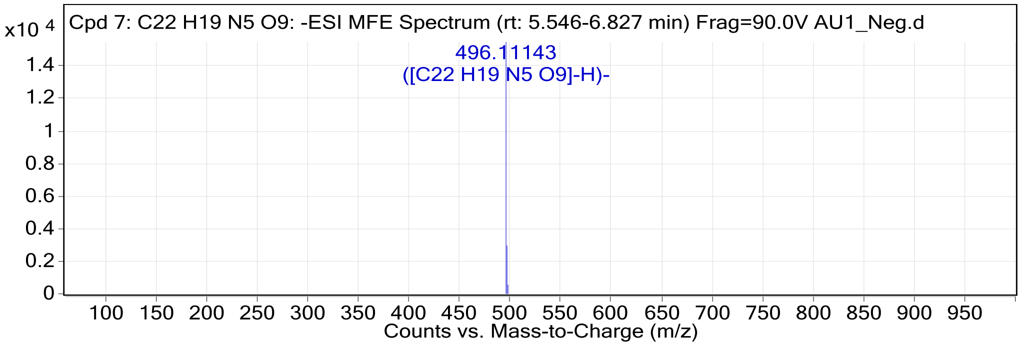

Peak List

m/z	z	Abund	Formula	Ion
496.11143	-1	15467.56	C22 H19 N5 O9	(M-H)-
497.1152	-1	2967.32	C22 H19 N5 O9	(M-H)-
498.11525	-1	561.46	C22 H19 N5 O9	(M-H)-

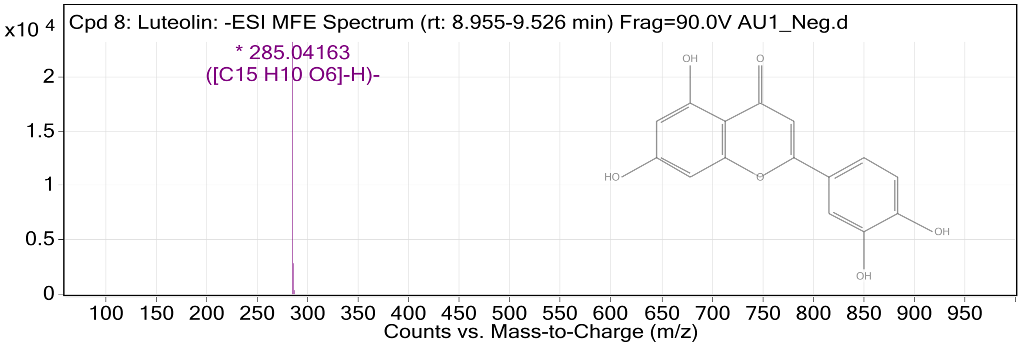

Peak List

m/z	z	Abund	Name	Formula	Ion	Score (DB)	Hits (DB)
285.04163	-1	23230.36	Luteolin	C15 H10 O6	(M-H)-	89.55	10
286.04555	-1	2838.9		C15 H10 O6	(M-H)-		

287.04712	-1	368.29		C15 H10 O6	(M-H)-		

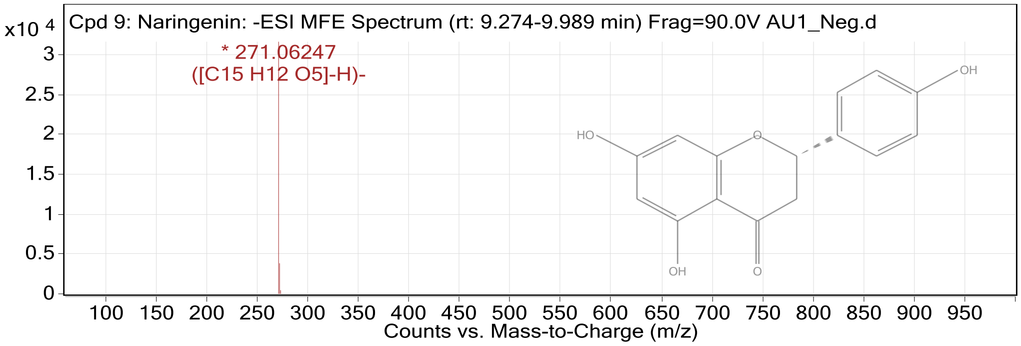

Peak List

m/z	z	Abund	Name	Formula	Ion	Score (DB)	Hits (DB)
271.06247	-1	31658.24	Naringenin	C15 H12 O5	(M-H)-	88.47	10
272.06633	-1	3841.21		C15 H12 O5	(M-H)-		
273.06761	-1	456.73		C15 H12 O5	(M-H)-		

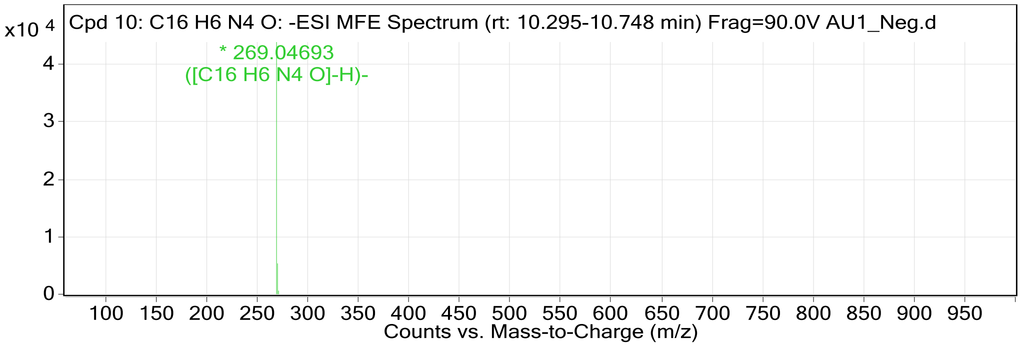

Peak List

m/z	z	Abund	Formula	Ion
269.04693	-1	43887.52	C16 H6 N4 O	(M-H)-
270.05078	-1	5365.06	C16 H6 N4 O	(M-H)-
271.05308	-1	623.27	C16 H6 N4 O	(M-H)-

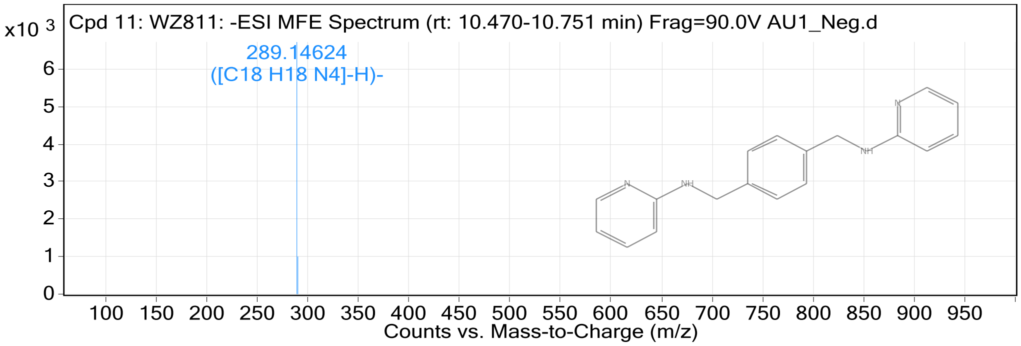

Peak List

m/z	z	Abund	Name	Formula	Ion	Score (DB)	Hits (DB)
289.14624	-1	6725.75	WZ811	C18 H18 N4	(M-H)-	78.78	1
290.14943	-1	1001.53		C18 H18 N4	(M-H)-		

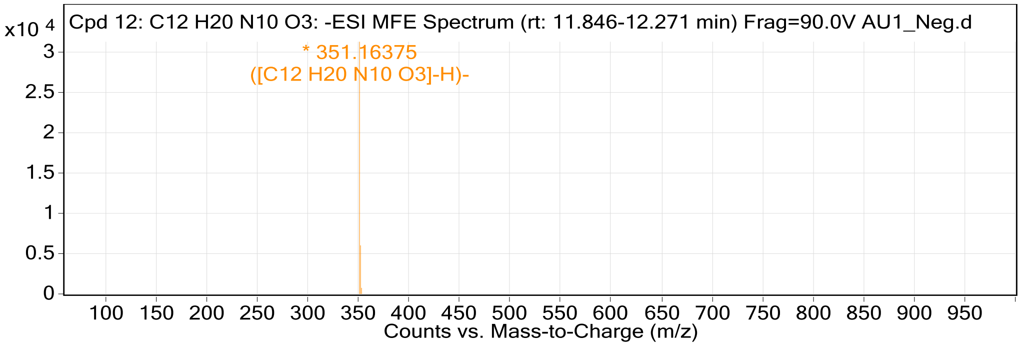

Peak List

m/z	z	Abund	Formula	Ion
351.16375	-1	31264.07	C12 H20 N10 O3	(M-H)-
352.16661	-1	6020.64	C12 H20 N10 O3	(M-H)-
353.16923	-1	751.64	C12 H20 N10 O3	(M-H)-

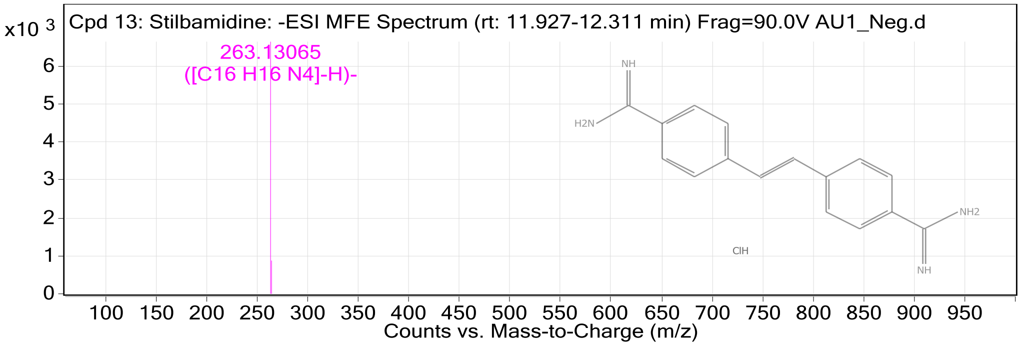

Peak List

m/z	z	Abund	Name	Formula	Ion	Score (DB)	Hits (DB)
263.13065	-1	6643.1	Stilbamidine	C16 H16 N4	(M-H)-	79.45	1
264.13344	-1	875.36		C16 H16 N4	(M-H)-		

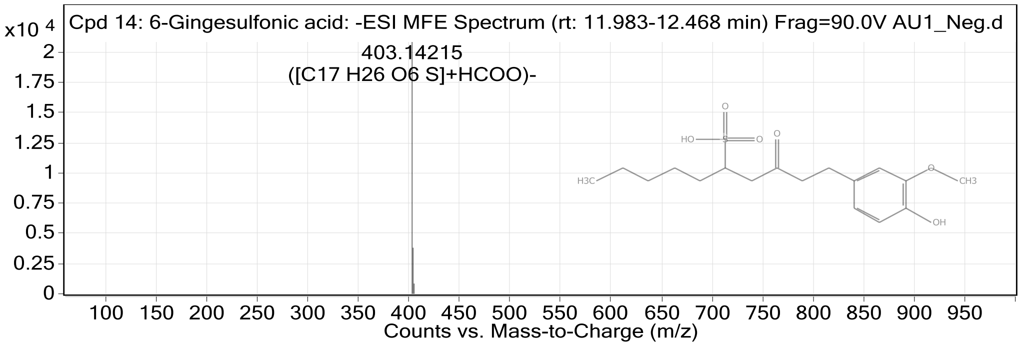

Peak List

m/z	z	Abund	Name	Formula	Ion	Score (DB)	Hits (DB)
403.14215	-1	20828.5	6-Gingesulfonic acid	C17 H26 O6 S	(M+HCOO)-	91.89	1
404.14627	-1	3816.97		C17 H26 O6 S	(M+HCOO)-		
405.144	-1	853.35		C17 H26 O6 S	(M+HCOO)-		

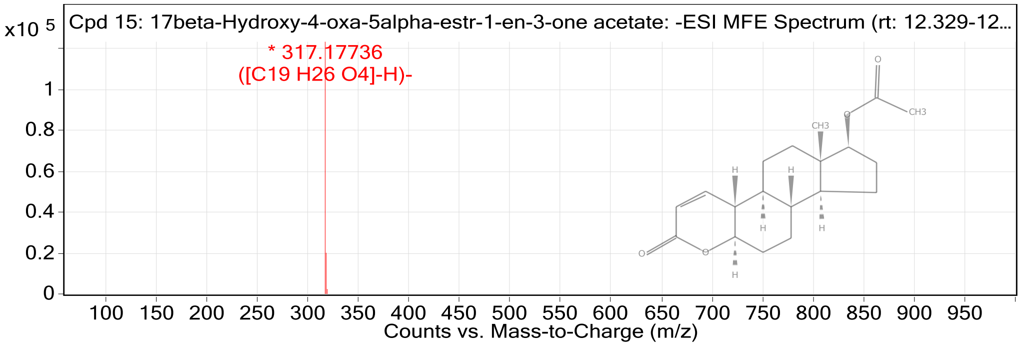

Peak List

m/z	z	Abund	Name	Formula	Ion	Score (DB)	Hits (DB)
317.17736	-1	123212.57	17beta-Hydroxy-4-oxa-5alpha-estr-1-en-3-one acetate	C19 H26 O4	(M-H)-	87.92	8
318.18087	-1	20125.4		C19 H26 O4	(M-H)-		
319.18417	-1	2409.68		C19 H26 O4	(M-H)-		

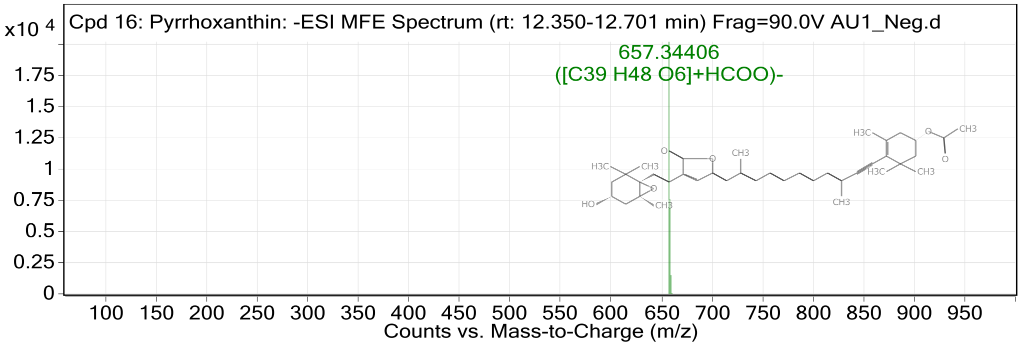

Peak List

m/z	z	Abund	Name	Formula	Ion	Score (DB)	Hits (DB)
657.34406	-1	20237.01	Pyrrhoxanthin	C39 H48 O6	(M+HCOO)-	92.58	1
658.34878	-1	7576.79		C39 H48 O6	(M+HCOO)-		
659.34972	-1	1507.46		C39 H48 O6	(M+HCOO)-		
660.34802	-1	71.76		C39 H48 O6	(M+HCOO)-		

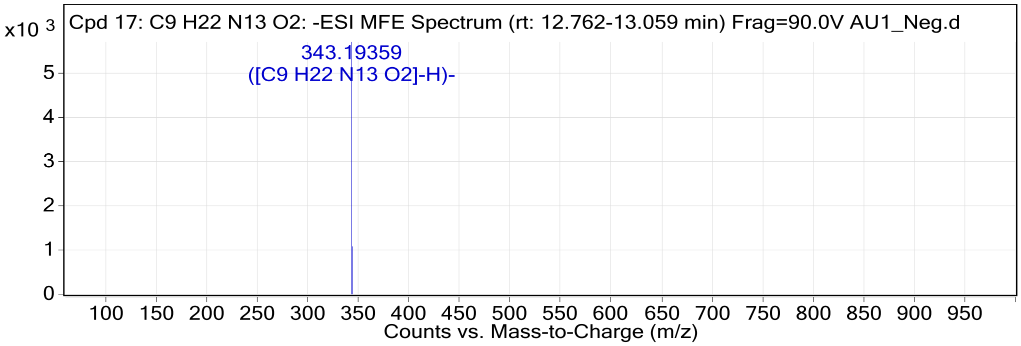

Peak List

m/z	z	Abund	Formula	Ion
343.19359	-1	5699.71	C9 H22 N13 O2	(M-H)-
344.1964	-1	1079.27	C9 H22 N13 O2	(M-H)-

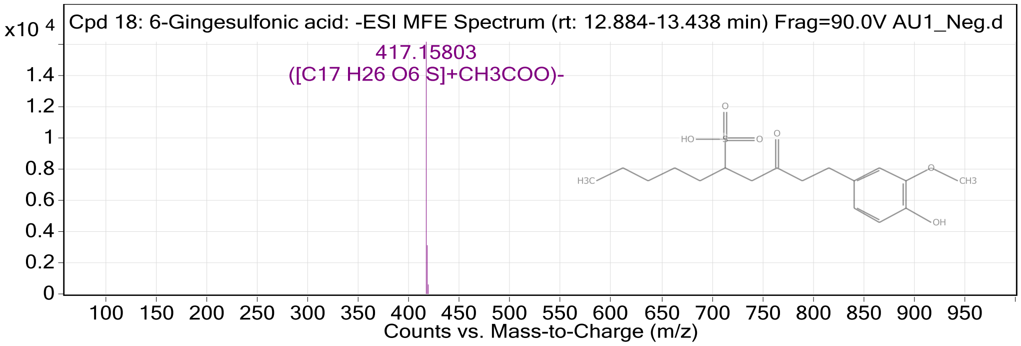

Peak List

m/z	z	Abund	Name	Formula	Ion	Score (DB)	Hits (DB)
417.15803	-1	16142.94	6-Gingesulfonic acid	C17 H26 O6 S	(M+CH3COO)-	91.76	1
418.16198	-1	3119.8		C17 H26 O6 S	(M+CH3COO)-		
419.1618	-1	609.08		C17 H26 O6 S	(M+CH3COO)-		

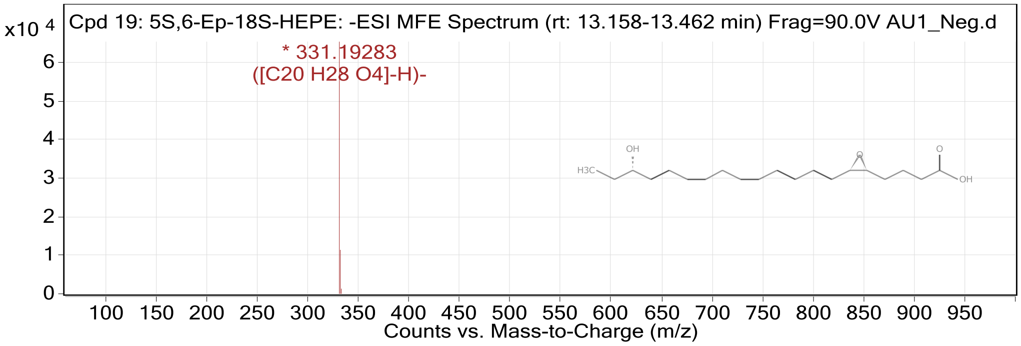

Peak List

m/z	z	Abund	Name	Formula	Ion	Score (DB)	Hits (DB)
331.19283	-1	65390.96	5S,6-Ep-18S-HEPE	C20 H28 O4	(M-H)-	89.51	10
332.19658	-1	11397.19		C20 H28 O4	(M-H)-		
333.19865	-1	1329.47		C20 H28 O4	(M-H)-		

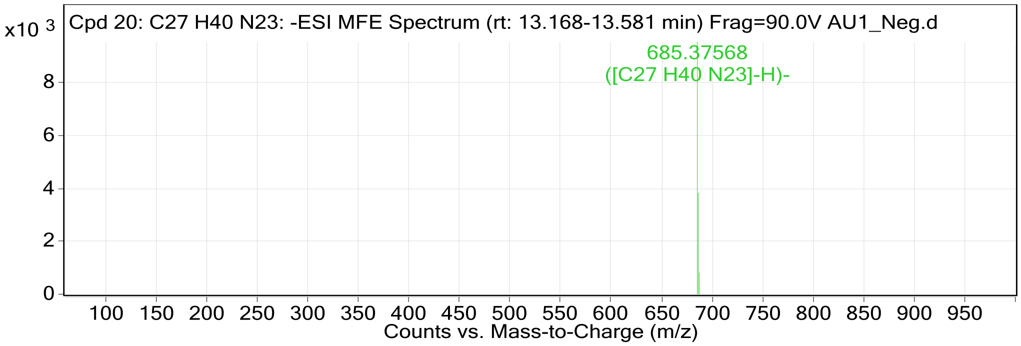

Peak List

m/z	z	Abund	Formula	Ion
685.37568	-1	9523.17	C27 H40 N23	(M-H)-
686.37882	-1	3833.63	C27 H40 N23	(M-H)-
687.37887	-1	819.81	C27 H40 N23	(M-H)-

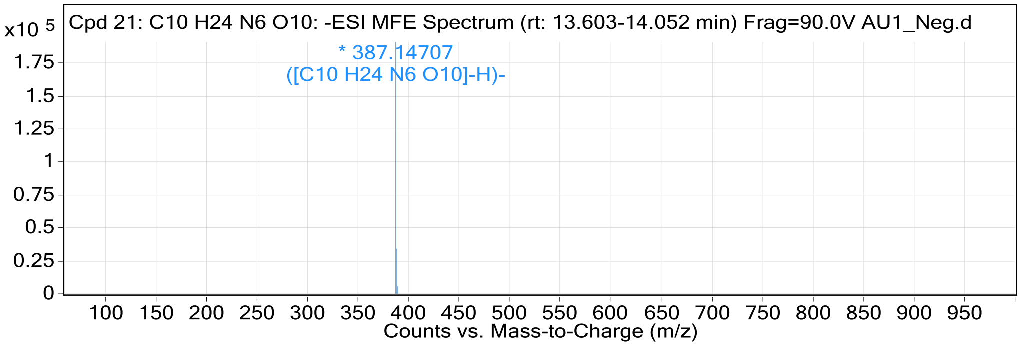

Peak List

m/z	z	Abund	Name	Formula	Ion	Score (DB)	Hits (DB)
387.14707	-1	190971.19	Bepotastine	C10 H24 N6 O10	(M-H)-	66.9	3
388.15013	-1	34198.03		C10 H24 N6 O10	(M-H)-		
389.15349	-1	5663.91		C10 H24 N6 O10	(M-H)-		
390.15499	-1	18.86		C10 H24 N6 O10	(M-H)-		

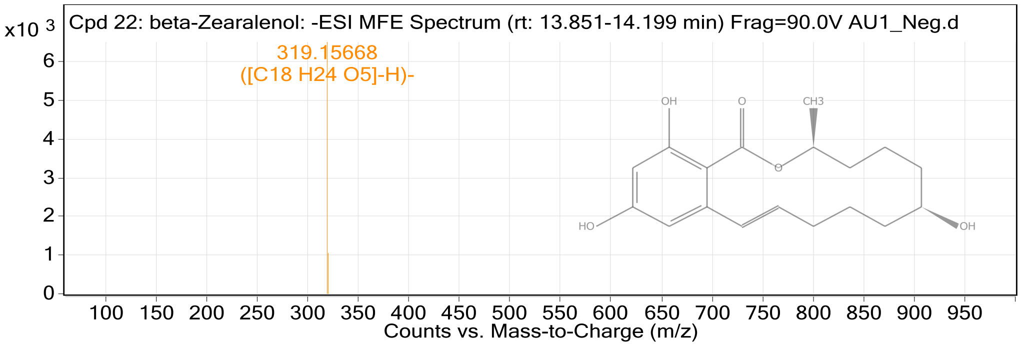

Peak List

m/z	z	Abund	Name	Formula	Ion	Score (DB)	Hits (DB)
319.15668	-1	6517.27	beta-Zearalenol	C18 H24 O5	(M-H)-	75.81	4
320.15981	-1	1057.8		C18 H24 O5	(M-H)-		

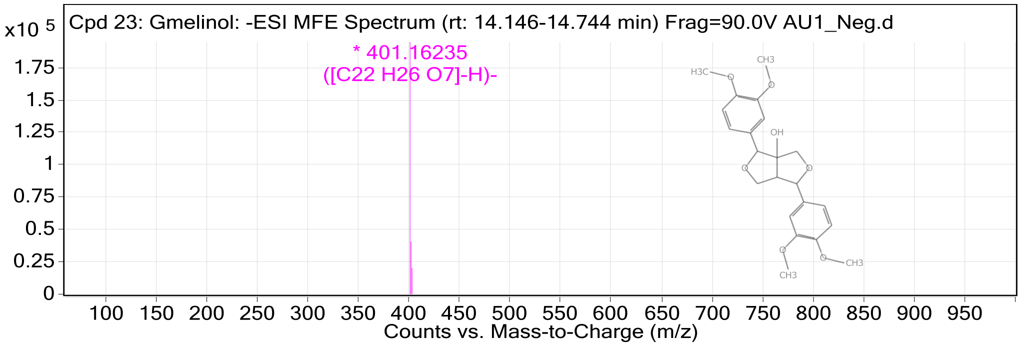

Peak List

m/z	z	Abund	Name	Formula	Ion	Score (DB)	Hits (DB)
401.16235	-1	194746.47	Gmelinol	C22 H26 O7	(M-H)-	78.15	10
402.16546	-1	40620.32		C22 H26 O7	(M-H)-		
403.15133	-1	20251.94		C22 H26 O7	(M-H)-		

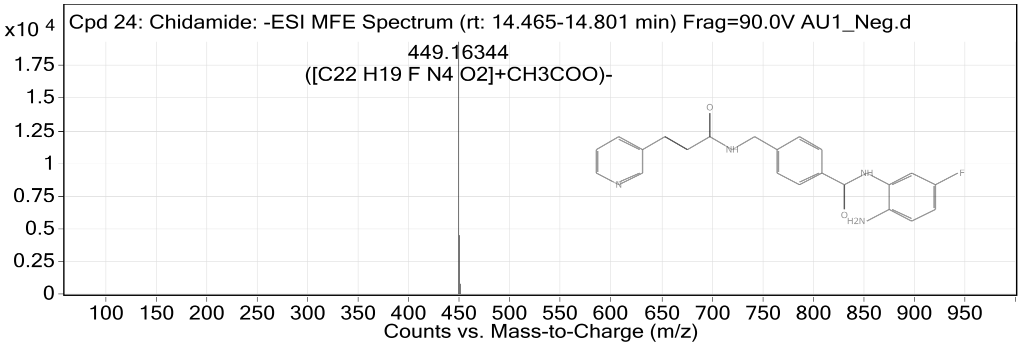

Peak List

m/z	z	Abund	Name	Formula	Ion	Score (DB)	Hits (DB)
449.16344	-1	19324.04	Chidamide	C22 H19 F N4 O2	(M+CH3COO)-	94.6	4
450.16758	-1	4498.11		C22 H19 F N4 O2	(M+CH3COO)-		
451.16913	-1	780.84		C22 H19 F N4 O2	(M+CH3COO)-		

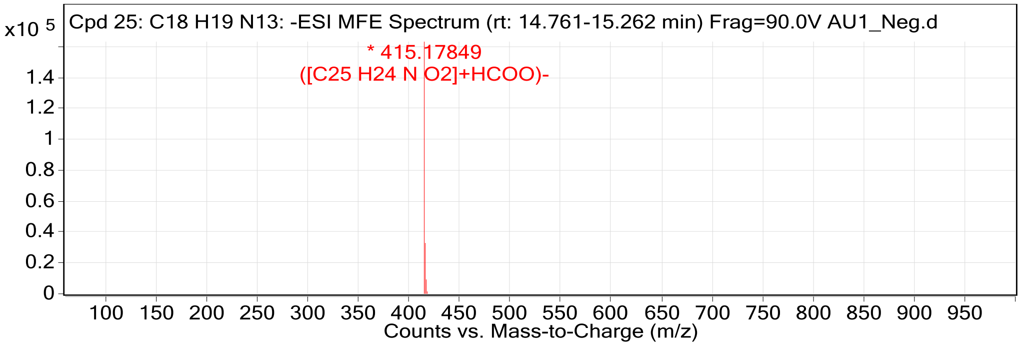

Peak List

m/z	z	Abund	Name	Formula	Ion	Score (DB)	Hits (DB)
415.17849	-1	163321.89	5β-Androstan-3α-ol-17-one sulfate	C25 H24 N O2	(M+HCOO)-	91.76	6
416.18146	-1	32947.17		C18 H19 N13	(M-H)-		
417.18411	-1	9323.14		C18 H19 N13	(M-H)-		
418.18554	-1	1562.56		C18 H19 N13	(M-H)-		

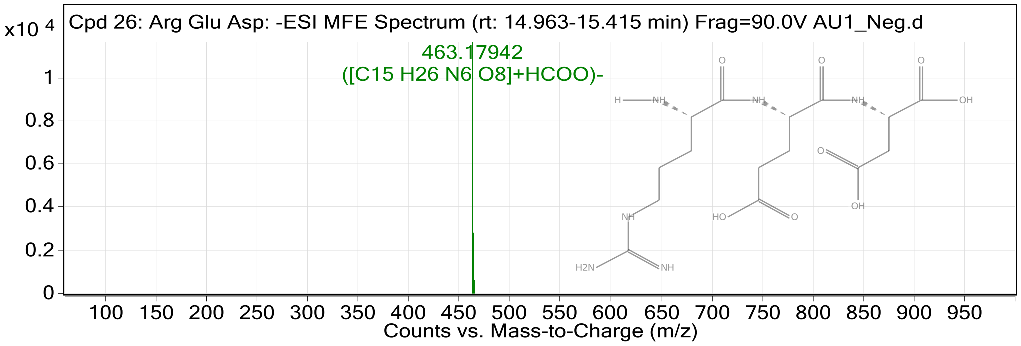

Peak List

m/z	z	Abund	Name	Formula	Ion	Score (DB)	Hits (DB)
463.17942	-1	11652.22	Arg Glu Asp	C15 H26 N6 O8	(M+HCOO)-	95.85	10
464.18298	-1	2818.7		C15 H26 N6 O8	(M+HCOO)-		
465.1843	-1	621.94		C15 H26 N6 O8	(M+HCOO)-		

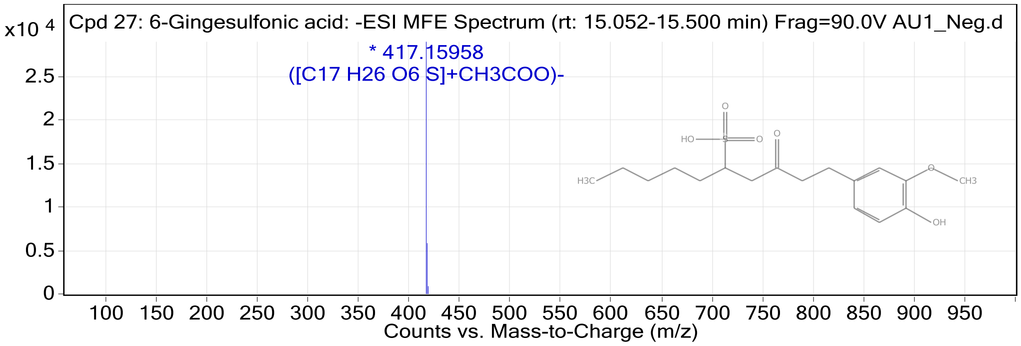

Peak List

m/z	z	Abund	Name	Formula	Ion	Score (DB)	Hits (DB)
417.15958	-1	29045.23	6-Gingesulfonic acid	C17 H26 O6 S	(M+CH3COO)-	90.29	1
418.1624	-1	5850.94		C17 H26 O6 S	(M+CH3COO)-		
419.16394	-1	892.72		C17 H26 O6 S	(M+CH3COO)-		

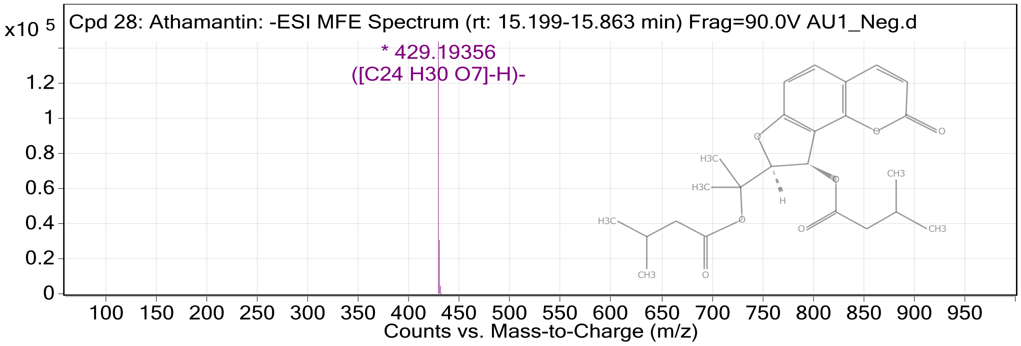

Peak List

m/z	z	Abund	Name	Formula	Ion	Score (DB)	Hits (DB)
429.19356	-1	143766.09	Athamantin	C24 H30 O7	(M-H)-	87.91	10
430.19714	-1	30714.02		C24 H30 O7	(M-H)-		
431.20198	-1	4621.35		C24 H30 O7	(M-H)-		
432.19885	-1	507.84		C24 H30 O7	(M-H)-		

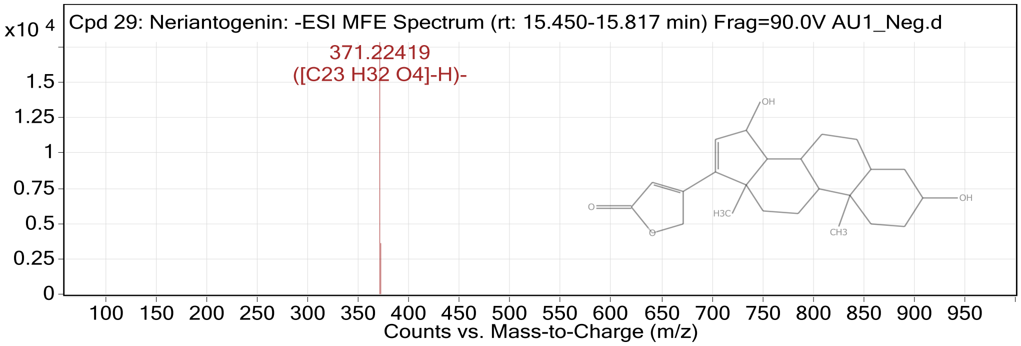

Peak List

m/z	z	Abund	Name	Formula	Ion	Score (DB)	Hits (DB)
371.22419	-1	17864.85	Neriantogenin	C23 H32 O4	(M-H)-	74.81	10
372.22797	-1	3614.48		C23 H32 O4	(M-H)-		

Qualitative Analysis Report

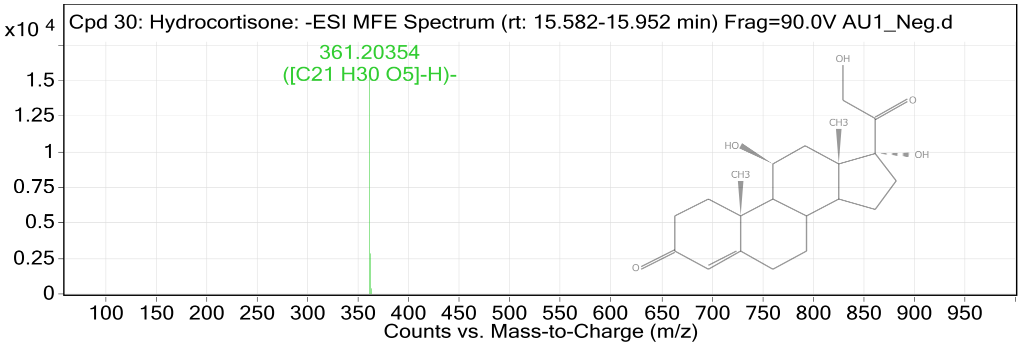

Peak List

m/z	z	Abund	Name	Formula	Ion	Score (DB)	Hits (DB)
361.20354	-1	15340.99	Hydrocortisone	C21 H30 O5	(M-H)-	89.35	10
362.20718	-1	2845.92		C21 H30 O5	(M-H)-		
363.20833	-1	387.22		C21 H30 O5	(M-H)-		

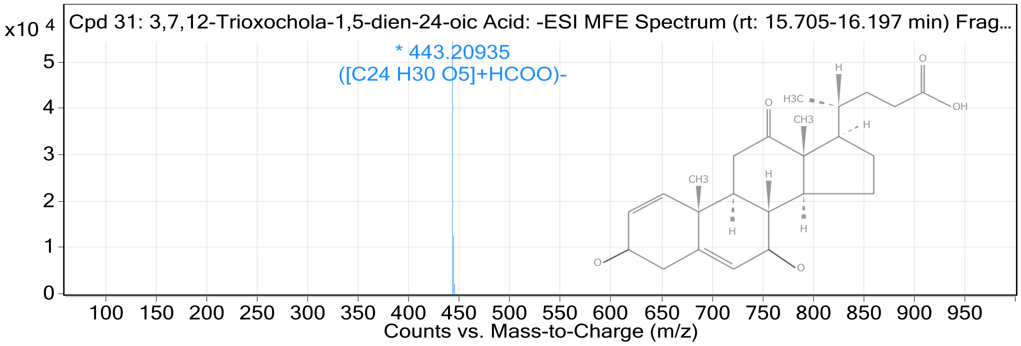

Peak List

m/z	z	Abund	Name	Formula	Ion	Score (DB)	Hits (DB)
443.20935	-1	54392.51	Sunitinib	C24 H30 O5	(M+HCOO)-	95.66	10
444.21312	-1	12599.05		C24 H30 O5	(M+HCOO)-		
445.21954	-1	2021.39		C24 H30 O5	(M+HCOO)-		
446.21001	-1	121.89		C24 H30 O5	(M+HCOO)-		

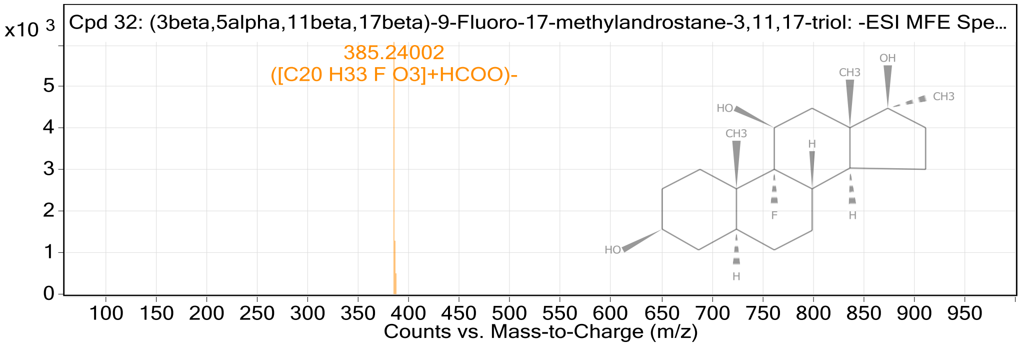

Peak List

m/z	z	Abund	Name	Formula	Ion	Score (DB)	Hits (DB)
385.24002	-1	6079.41	(3beta,5alpha,11beta,17beta)-9-Fluoro-17-methylandrostane-3,11,17-triol	C20 H33 F O3	(M+HCOO)-	81.25	10
386.24314	-1	1288.32		C20 H33 F O3	(M+HCOO)-		
387.22751	-1	502.48		C20 H33 F O3	(M+HCOO)-		

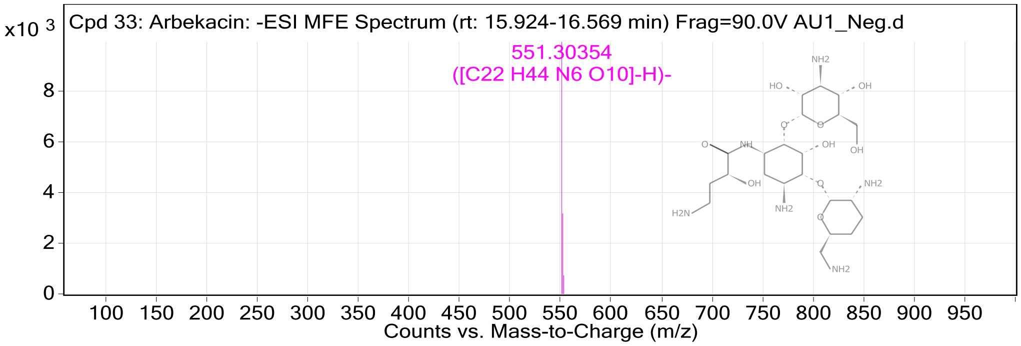

Peak List

m/z	z	Abund	Name	Formula	Ion	Score (DB)	Hits (DB)
551.30354	-1	9925.86	Triterpenoid	C22 H44 N6 O10	(M-H)-	92.95	3
552.3069	-1	3168.91		C22 H44 N6 O10	(M-H)-		
553.30552	-1	735.12		C22 H44 N6 O10	(M-H)-		

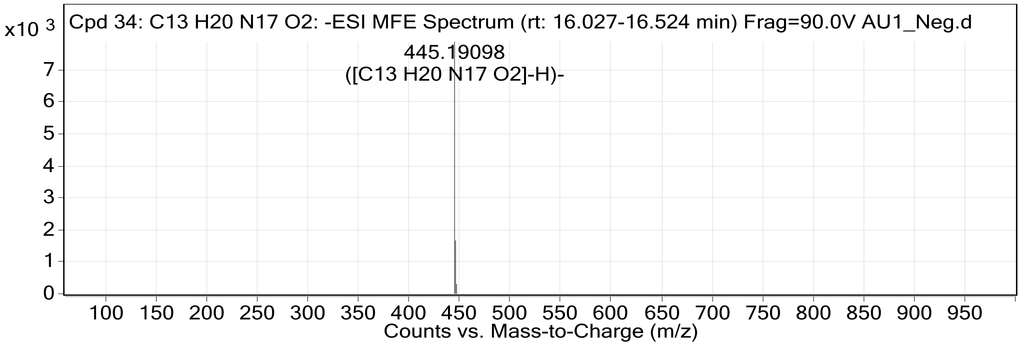

Peak List

m/z	z	Abund	Name	Formula	Ion	Score (DB)	Hits (DB)
445.19098	-1	7880.15	Ptilosteroid A	C13 H20 N17 O2	(M-H)-	90.66	1
446.19289	-1	1668		C13 H20 N17 O2	(M-H)-		
447.19332	-1	305.65		C13 H20 N17 O2	(M-H)-		

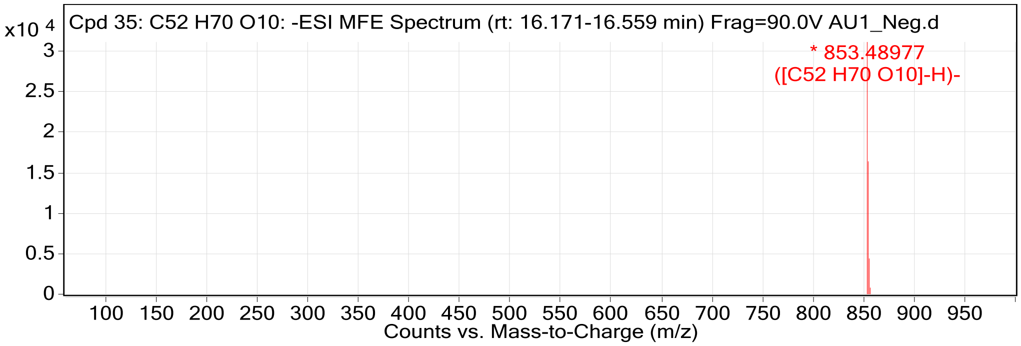

Peak List

m/z	z	Abund	Name	Formula	Ion	Score (DB)	Hits (DB)
853.48977	-1	31125.46	PI(18:3(6Z,9Z,12Z)/18:3(9Z,12Z,15Z))	C52 H70 O10	(M-H)-	90.23	10
854.49417	-1	16397.88		C52 H70 O10	(M-H)-		
855.49722	-1	4410.1		C52 H70 O10	(M-H)-		
856.49618	-1	813.93		C52 H70 O10	(M-H)-		

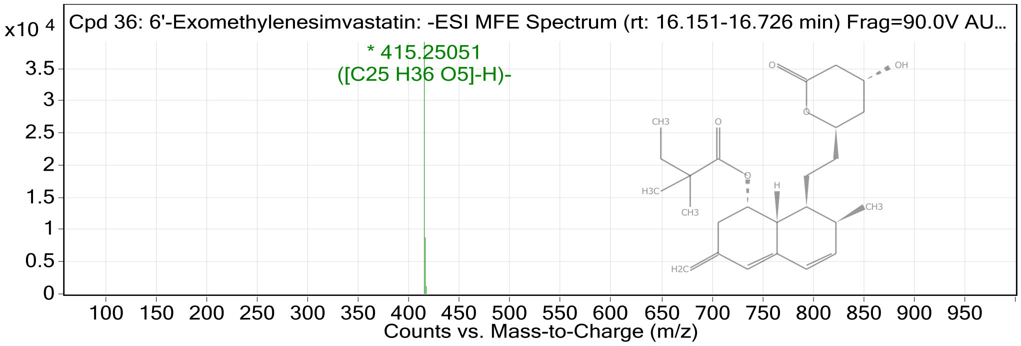

Peak List

m/z	z	Abund	Name	Formula	Ion	Score (DB)	Hits (DB)
415.25051	-1	39182.33	6'-Exomethylenesimvastatin	C25 H36 O5	(M-H)-	88.57	10
416.2546	-1	8790.86		C25 H36 O5	(M-H)-		
417.25713	-1	1219.72		C25 H36 O5	(M-H)-		

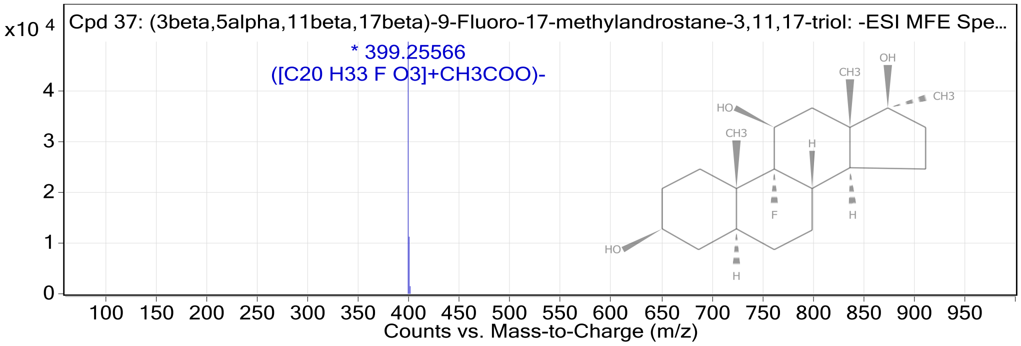

Peak List

m/z	z	Abund	Name	Formula	Ion	Score (DB)	Hits (DB)
399.25566	-1	49739.46	[illegible] 9-Fluoro-17-methylandrostane-3,11,17-triol	C20 H33 F O3	(M+CH3COO)-	98.29	10
400.25954	-1	11268.51		C20 H33 F O3	(M+CH3COO)-		
401.26155	-1	1513.1		C20 H33 F O3	(M+CH3COO)-		

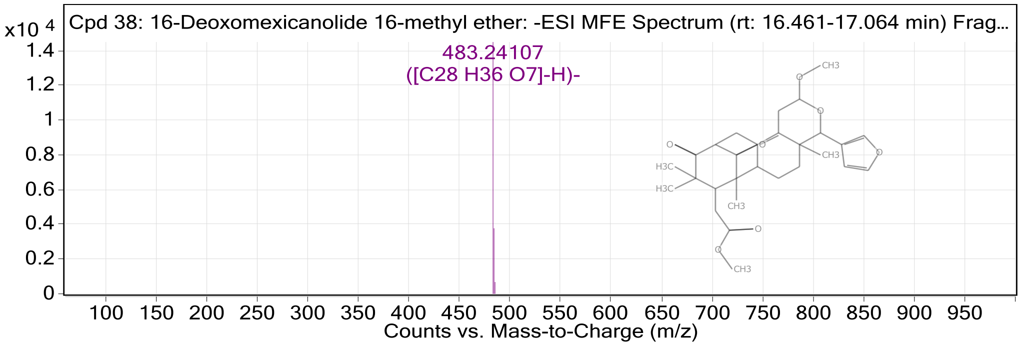

Peak List

m/z	z	Abund	Name	Formula	Ion	Score (DB)	Hits (DB)
483.24107	-1	14486.96	16-Deoxomexicanolide 16-methyl ether	C28 H36 O7	(M-H)-	86.47	5
484.24478	-1	3771.24		C28 H36 O7	(M-H)-		
485.24563	-1	679.39		C28 H36 O7	(M-H)-		

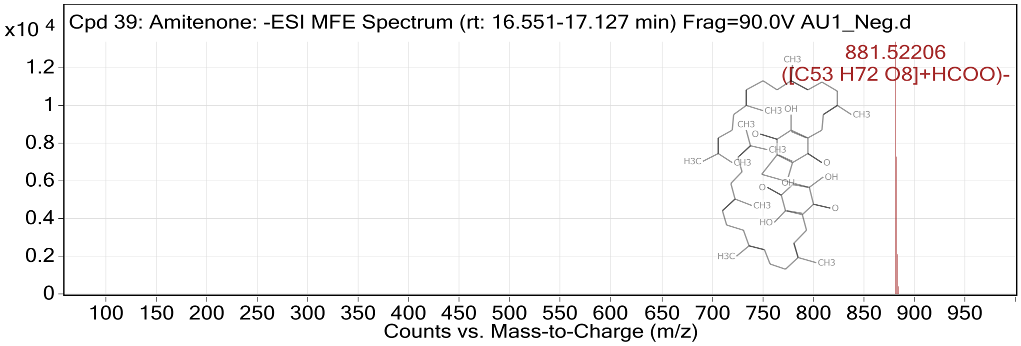

Peak List

m/z	z	Abund	Name	Formula	Ion	Score (DB)	Hits (DB)
881.52206	-1	13401.67	Amitenone	C53 H72 O8	(M+HCOO)-	96.38	10
882.52566	-1	7300.34		C53 H72 O8	(M+HCOO)-		
883.52615	-1	2107.56		C53 H72 O8	(M+HCOO)-		
884.52487	-1	401.9		C53 H72 O8	(M+HCOO)-		

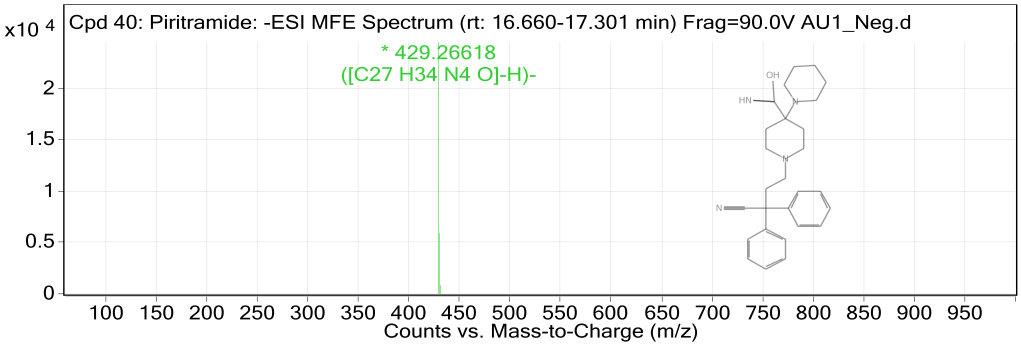

Peak List

m/z	z	Abund	Name	Formula	Ion	Score (DB)	Hits (DB)
429.26618	-1	24543.4	Piritramide	C27 H34 N4 O	(M-H)-	91.8	10
430.27033	-1	5950.48		C27 H34 N4 O	(M-H)-		
431.27129	-1	862.78		C27 H34 N4 O	(M-H)-		

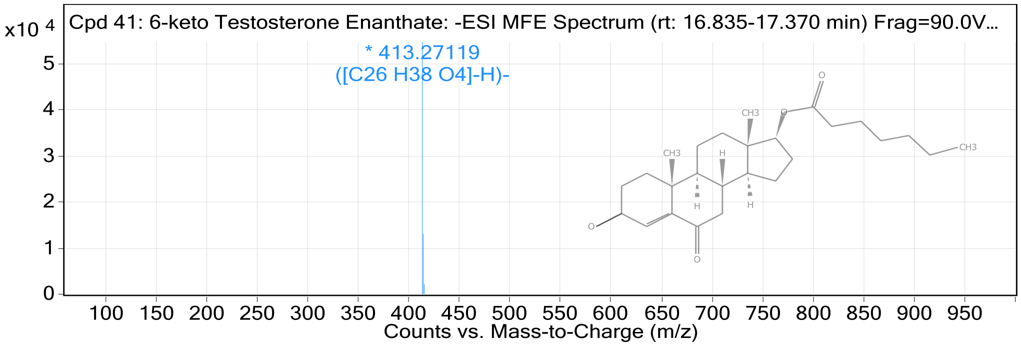

Peak List

m/z	z	Abund	Name	Formula	Ion	Score (DB)	Hits (DB)
413.27119	-1	54805	6-keto Testosterone Enanthate	C26 H38 O4	(M-H)-	90.29	10
414.27513	-1	13072.37		C26 H38 O4	(M-H)-		
415.2754	-1	2186.57		C26 H38 O4	(M-H)-		

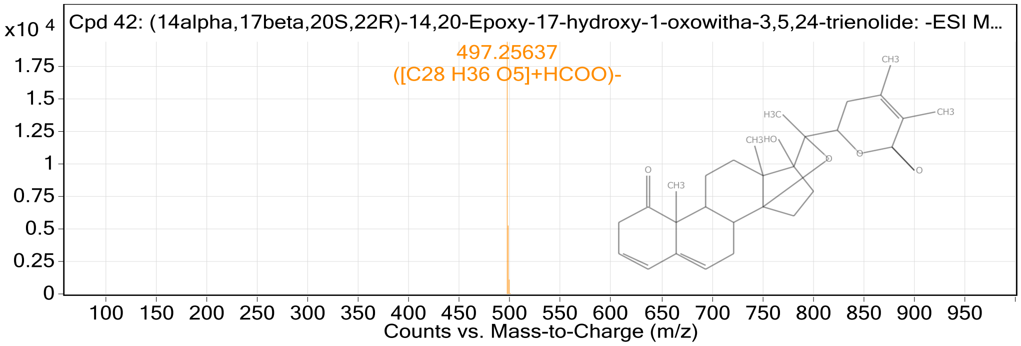

Peak List

m/z	z	Abund	Name	Formula	Ion	Score (DB)	Hits (DB)
497.25637	-1	19433.94	(14alpha,17beta,20S,22R)-14,20-Epoxy-17-hydroxy-1-oxowitha-3,5,24-trienolide	C28 H36 O5	(M+HCOO)-	86.21	3
498.26025	-1	5273.18		C28 H36 O5	(M+HCOO)-		
499.25595	-1	1106.79		C28 H36 O5	(M+HCOO)-		
500.25465	-1	64.94		C28 H36 O5	(M+HCOO)-		

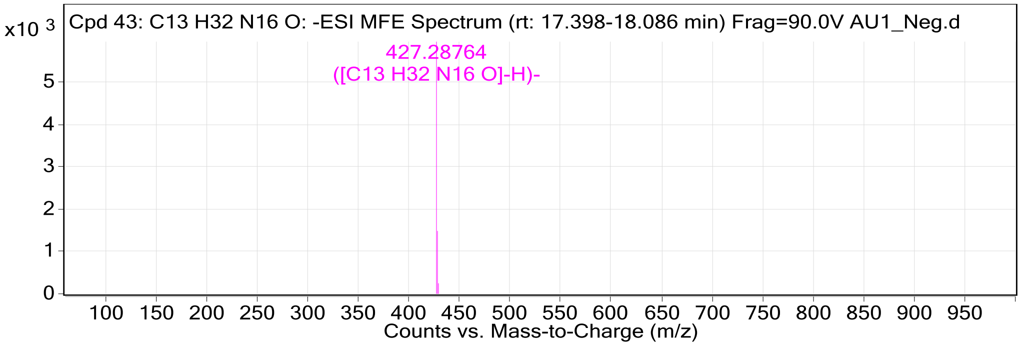

Peak List

m/z	z	Abund	Formula	Ion
427.28764	-1	5956.09	C13 H32 N16 O	(M-H)-
428.29058	-1	1484.66	C13 H32 N16 O	(M-H)-
429.27759	-1	249.06	C13 H32 N16 O	(M-H)-

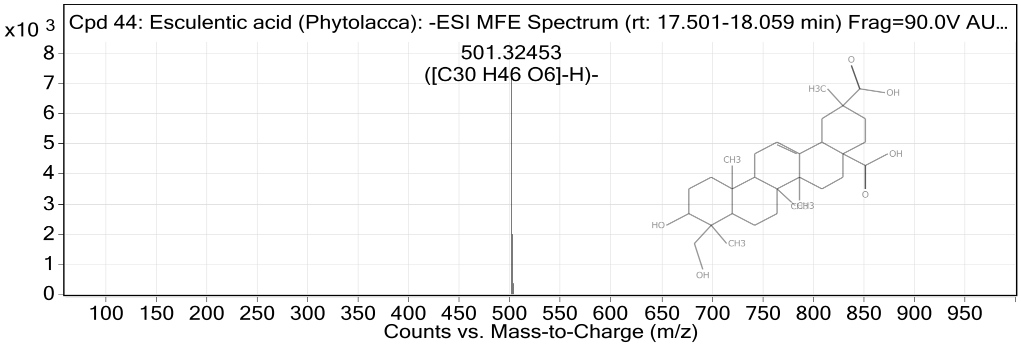

Peak List

m/z	z	Abund	Name	Formula	Ion	Score (DB)	Hits (DB)
501.32453	-1	7271.06	Esculentic acid (Phytolacca)	C30 H46 O6	(M-H)-	85.51	5
502.32783	-1	1994.09		C30 H46 O6	(M-H)-		
503.32758	-1	366.18		C30 H46 O6	(M-H)-		

--- End Of Report ---
